# Restriction of influenza A virus replication by host DCAF7-CRL4B axis

**DOI:** 10.1128/jvi.00133-25

**Published:** 2025-03-27

**Authors:** Lei Yu, Yong Jiang, Hongyu Rang, Xueyun Wang, Yumeng Cai, Haojie Yan, Shuwen Wu, Ke Lan

**Affiliations:** 1 Frontier Science Center for Immunology and Metabolism, Medical Research Institute, Wuhan University 619779, Wuhan, China; 2 State Key Laboratory of Virology and Biosafety, College of Life Sciences, Wuhan University 98436 , Wuhan, China; 3 Taikang Center for Life and Medical Sciences, Wuhan University, Wuhan, China; St. Jude Children's Research Hospital, Memphis, Tennessee, USA

**Keywords:** influenza A virus, DCAF7, CRL4B, PA, proteasomal degradation

## Abstract

The balance between cellular defense and viral escape determines the fate of influenza A virus (IAV) infection. Viral polymerase activity is critical for the replication and propagation of IAV. The antiviral strategies of host cells against IAV infection have not been fully elucidated. Here, we identified DCAF7 as an antiviral factor for IAV, which inhibits the replication of H1N1 and H3N2. Mechanistically, DCAF7 weakens the viral heterotrimer polymerase activity and restricts IAV replication and transcription. DCAF7 as a substrate recognition receptor forms a complete CRL4B^DCAF7^ E3 ligase with the CRL4B E3 complex to promote K48-linked polyubiquitination of the viral polymerase subunit PA at the K609 site and its degradation. We also showed that a specific cullin-RING E3 ligase (CRL) inhibitor MLN4924 upregulates the protein level of PA and promotes the replication of IAV *in vivo*. Moreover, activation of CUL4B by etoposide promotes the degradation of PA and inhibits IAV replication *in vivo*. Importantly, we found that viral NS1 protein decreases DCAF7 level to impair its antiviral efficacy. Taken together, these findings reveal a new mechanism of host resistance to IAV infection and suggest that regulation of the DCAF7-CRL4B axis is a potential target for antivirals.

**IMPORTANCE:**

Until now, the key host factors that affect IAV polymerase have not been fully elucidated. In this study, we identified host DCAF7 as a novel restriction factor for IAV replication. Importantly, DCAF7 acts as a substrate recognition receptor to recruit CRL4B E3 ligase to mediate the degradation of PA through the ubiquitin-proteasome pathway. Further exploration demonstrated that a specific cullin-RING E3 ligase inhibitor MLN4924 promotes IAV replication *in vivo*, and activation of CUL4B by etoposide inhibits IAV replication *in vivo*. Notably, we found that the viral NS1 protein decreases DCAF7 level to impair its antiviral efficacy. These findings elucidate the critical function and mechanism of the DCAF7-CRL4B axis in IAV replication, reveal a novel host anti-IAV mechanism, and provide new anti-influenza drug development strategies.

## INTRODUCTION

Influenza A virus (IAV) is an important human viral pathogen that initiates seasonal epidemics and represents a considerable burden to public health with its substantial-high morbidity and mortality (1). Influenza viruses are classified into four types (A, B, C, and D), and two influenza A virus subtypes (H1N1 and H3N2) are mainly responsible for seasonal influenza epidemics, affecting 5%–15% of the population and causing 290,000 to 650,000 deaths every year (2, 3). Vaccines and antiviral drugs are commonly used to prevent and treat influenza. To date, although vaccines and antiviral drugs have made great progress, the prevention and treatment of influenza virus infection remain difficult due to its continuous mutations and numerous viral subtypes (1, 4). The vaccine only prevents the infection of known subtypes of influenza virus and is ineffective for the new influenza strains produced by antigenic drift or antigenic shift (5). Antiviral drugs that target viral proteins lose their antiviral activity due to drug resistance. More importantly, the emergence of new pandemic strains is sudden and unpredictable, and vaccination and existing antiviral drugs are not able to quickly and effectively control the spread of diseases (6, 7). Hence, a deeper exploration of viral replication and host-virus regulation to provide novel cellular targets for broad-spectrum anti-influenza drug development is urgently needed.

Influenza A virus belongs to the *orthomyxoviridae* family and is an enveloped particle with a segmented, negative-sense RNA genome (8). The viral ribonucleoprotein (vRNP) is composed of viral RNA, multiple nucleoproteins (NPs), and one RNA-dependent RNA polymerase (RdRp) complex, which is responsible for the replication and transcription of the IAV genome in the nucleus. RdRp is a trimeric complex containing two basic proteins, polymerase basic protein 1 (PB1) and polymerase basic protein 2 (PB2), and a polymerase acidic protein (PA) (9, 10). PB1 is mainly responsible for the RNA elongation and the addition of 3′ poly A tails to the RNA ends, while PB2 binds to m7G on host RNA by cap-binding domain, and PA with endonuclease activity cuts the mRNA 5′ cap structure of host cells as a primer for viral transcription (11–14). These three subunits of RdRp are highly conserved among influenza A, B, and C viruses, and polymerase activity is critical for the proliferation and propagation of IAV. Therefore, it is promising to explore the key host factors that regulate the activity of polymerase and use them as targets for developing broad-spectrum drugs with low drug resistance. Currently, several studies have reported the identification of host factors that affect polymerase activity. SERTAD3 inhibits influenza A virus replication by blocking the assembly of the viral RNA polymerase complex, and 8-amino acid peptides derived from SERTAD3 effectively inhibit IAV infection (15). BAG6 promotes K48-linked polyubiquitination of PB2 at K189 and its degradation, thereby inhibiting viral polymerase activity (16). Galectin-3 enhances IAV replication by promoting RdRp activity through association with PA protein (17). TRIM32 limits viral infection by interacting with PB1 to induce its ubiquitination (18). However, the host factors that affect viral polymerase have not been fully elucidated, especially the host proteins that directly target PA subunits for degradation are rarely reported.

Here, we identified host DCAF7 as a negative regulator of the influenza A virus. DCAF7 (DDB1 and CUL4-associated factor 7) also known as WDR68, is a highly conserved member of the WD40 repeat domain family and functions as a scaffolding protein with five WD40 domains (19). It was originally identified in DDB1 complexes and inferred to function as a substrate receptor in the cullin4-RING (CRL4) E3 ligases (20). Subsequent studies have revealed that DCAF7 is involved in various physiological and biochemical processes, including protein stability (21, 22), cell proliferation (23), nucleotide excision repair (24), cancer progression (25, 26), and development and differentiation (27, 28). In addition, two previous reports showed that DCAF7 interacts with both adenovirus E1A and human papillomavirus E6 protein (29, 30). However, the function of DCAF7 in IAV infection has not been determined.

In the present study, we identified DCAF7 as a negative regulator of IAV to restrict its replication. Mechanically, DCAF7 acts as a substrate recognition receptor to form a complete CRL4B^DCAF7^ E3 ligase with the CRL4B E3 complex to promote K48-linked polyubiquitination of IAV-PA at the K609 site and its degradation, thereby inhibiting viral polymerase activity and RNA synthesis and weakening IAV replication. We employed a specific cullin-RING E3 ligase (CRL) inhibitor, MLN4924. Treatment with MLN4924 upregulated the PA protein level and promoted the replication of IAV *in vivo*. Furthermore, activation of CUL4B by etoposide promoted the PA degradation and inhibited the IAV virulence *in vivo*. We also demonstrated that in IAV infection, viral NS1 protein downregulated the expression of DCAF7 to weaken its antiviral efficacy, which is beneficial to viral self-replication. Taken together, these findings are the first to identify PA ubiquitination-modified E3 ligase and reveal that the DCAF7-CRL4B axis can serve as an antiviral target for IAV.

## RESULTS

### Identification of DCAF7 as an antiviral factor against influenza A virus

**Fig 1:**
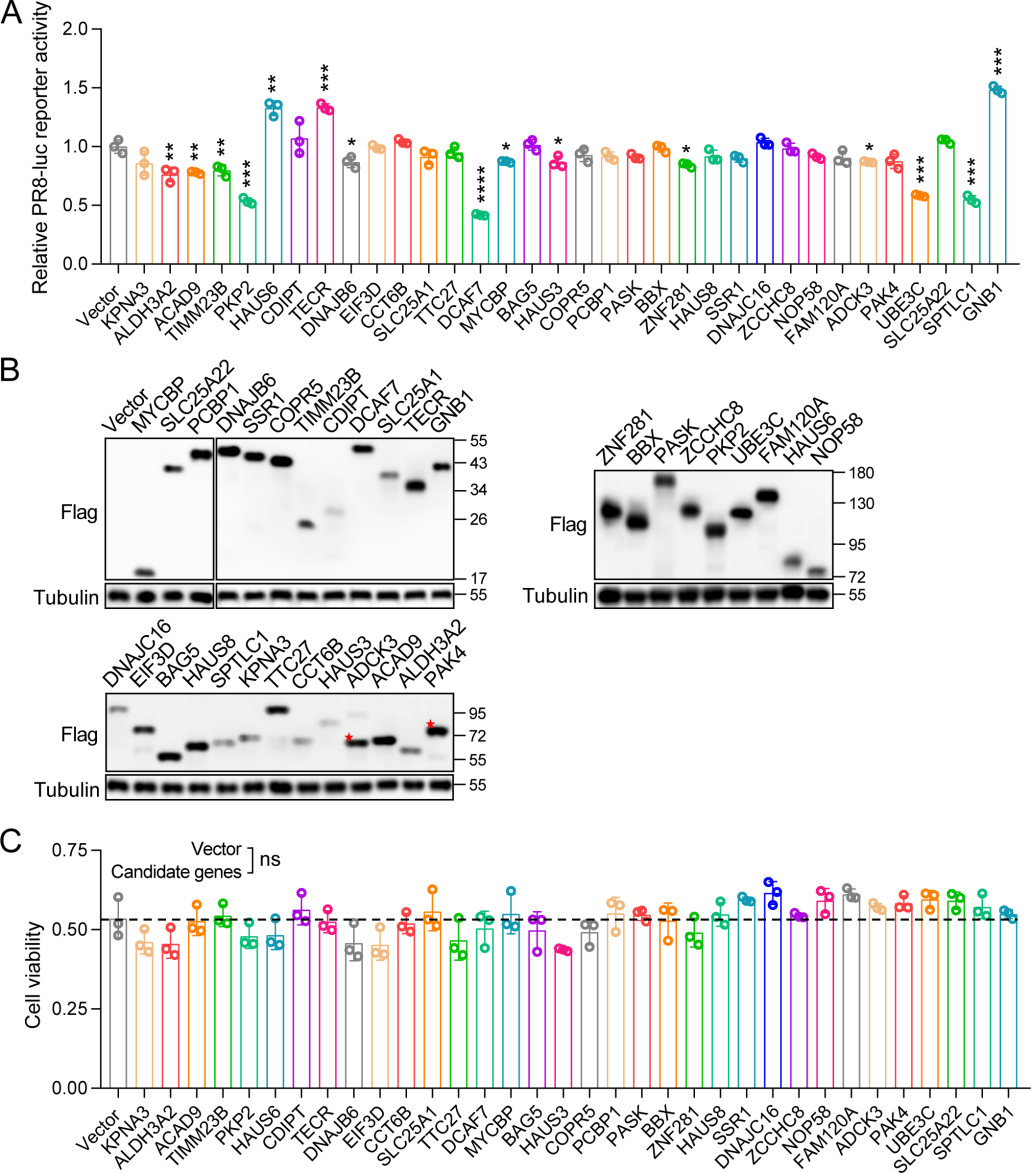
DCAF7 was identified to negatively regulate the infection of influenza A virus. (A and B) MDCK cells were transfected with the indicated plasmids (0.5 µg each), and then infected with PR8-Rluc virus at an MOI of 0.01 for 24 h, and then the luciferase activity was measured (A), and the expression of candidate genes was detected by Western blotting (B). (C) MDCK cells transfected with the indicated plasmids were measured for cell viability by CCK-8 assay. An unpaired t-test was used for data statistical analysis, and the data were shown as mean ± SD from three independent experiments. ns, no significance, *P < 0.05, **P < 0.01, ***P < 0.001, ****P < 0.0001.

Previous studies have identified many host factors that potentially interact with IAV viral proteins by mass spectrometry, and bioinformatics processing has reduced false positives and yielded high-confidence candidate proteins (31). To discover new host factors that directly regulate IAV polymerase subunits and affect IAV replication, 32 host proteins that potentially interact with IAV polymerase subunits were cloned and overexpressed based on the above mass spectrometry results. The function of 32 candidate host proteins in IAV infection has not been reported. Each candidate gene expression plasmid with Flag tag was transfected into MDCK cells, the cells were infected with PR8-Rluc virus 24 hours post-transfection, and the luciferase activity was measured 24 hours post-infection. Results showed that overexpression of DCAF7 significantly reduced IAV replication compared with the control group (Fig. 1A and B). Previous reports confirmed that GNB1 promotes PB2 nuclear import to enhance the replication of H1N1 and H9N2 (32), while PKP2 competes with PB2 for PB1 binding to restrict IAV replication (31). Using GNB1 and PKP2 as positive controls in the above infection experiment, our results also showed the same function of these two host factors in IAV replication, thus demonstrating the reliability of the experimental infection system. Furthermore, there was no significant change in cell viability 24 hours post-transfection of candidate genes (Fig. 1C). In summary, we found that DCAF7 is an overall antiviral factor against IAV.

### DCAF7 inhibits influenza A virus replication

**Fig 2:**
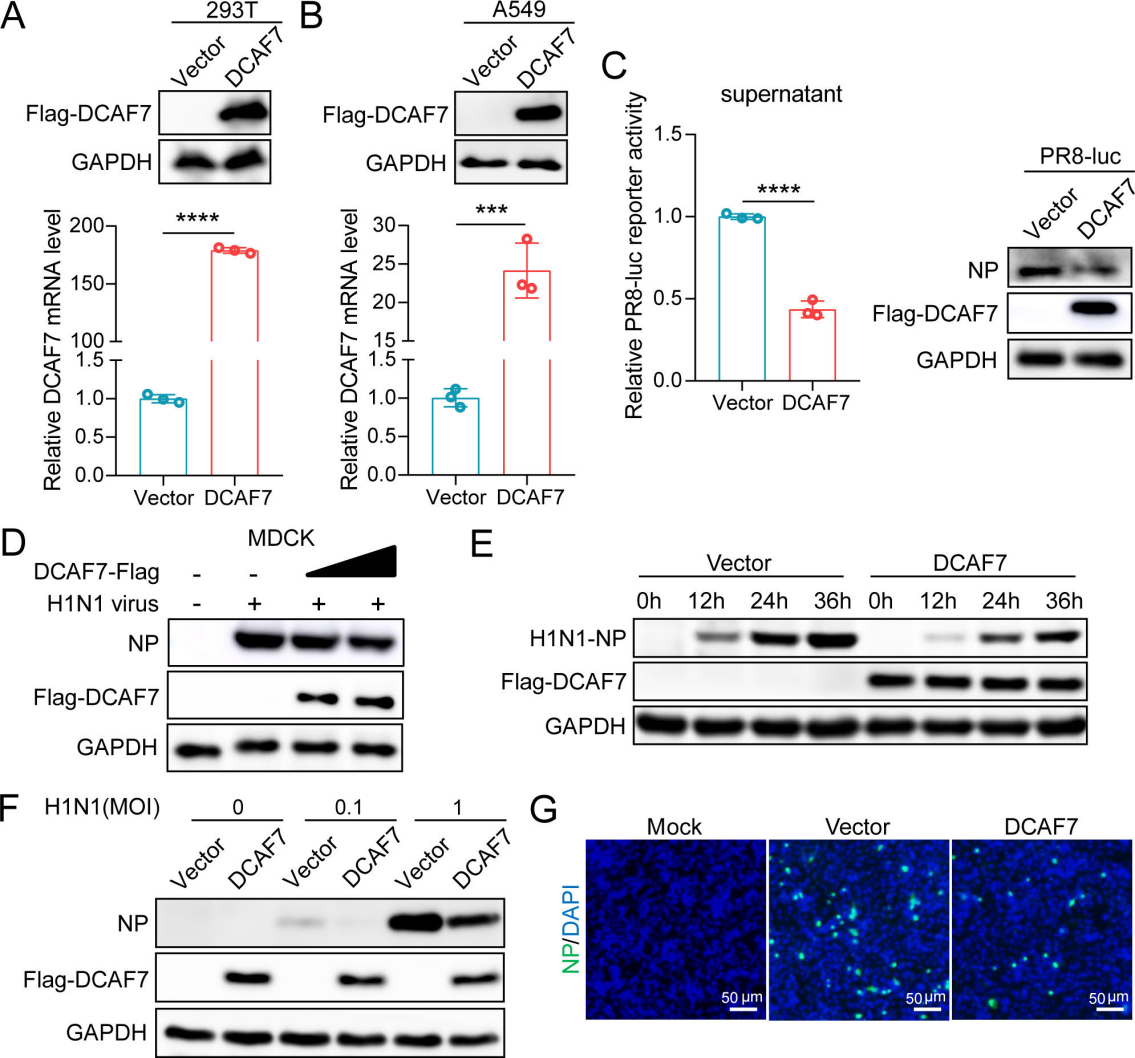
DCAF7 inhibits influenza A virus replication. (A and B) Construction of DCAF7 stable overexpressing cell line. HEK293T cells (A) or A549 cells (B) were stably transfected with lentiviruses containing a Flag-tagged DCAF7 expression plasmid or an empty vector plasmid, named 293T-DCAF7/293T-Vector cells and A549-DCAF7/A549-Vector cells, respectively. DCAF7 overexpression was verified by Western blotting and qRT-PCR. (C) 293T-DCAF7/293T-Vector cells were infected with PR8-Rluc virus for 48 h, and then the luciferase activity of the supernatants was measured (left), and the level of viral NP protein in the cells was detected by Western blotting (right). (D) MDCK cells were transfected with flag-tagged DCAF7 protein expression plasmid (0, 0.5, and 1 µg), and the viral NP protein level was examined after 24 h infection with H1N1 at an MOI of 0.01. (E) A549-DCAF7/A549-Vector cells were infected with H1N1 at an MOI of 0.1, and the viral NP protein level was examined at the indicated time points. (F) 293T-DCAF7/293T-Vector cells were infected with H1N1 (MOI = 0, 0.1, and 1) for 24 h. The viral NP protein level was examined by Western blotting. (G) A549-DCAF7/A549-Vector cells were infected with H1N1 at an MOI of 0.1 for 24 h. An immunofluorescence assay was performed and then visualized by fluorescence microscopy (NP: green; Nuclear: blue), scale bar = 50 µm. An unpaired t-test was used for data statistical analysis, and the data were shown as mean ± SD from three independent experiments. ***P < 0.001, ****P < 0.0001.

Since DCAF7 is a potential viral polymerase binding protein, we speculated that it may inhibit IAV by affecting its replication. To evaluate the antiviral effect of DCAF7 on IAV replication, we successfully constructed two lentiviral vector-based HEK293T and A549 cell lines stably expressing DCAF7. Quantitative RT-PCR and Western blotting analysis demonstrated that DCAF7 was overexpressed in these cells compared to the empty vector control cells (Fig. 2A and B). The control and DCAF7-expressing HEK293T cells were infected with the PR8-*Renilla* luciferase reporter virus, and then the luciferase activity of supernatants and the viral NP protein level in cell lysates were measured. The result showed that the expression of DCAF7 significantly inhibited IAV replication (Fig. 2C). We further detected the IAV NP protein level by Western blotting and immunofluorescence analysis to determine the role of DCAF7 on H1N1/H3N2 virus replication using three cell lines. First, MDCK cells were transfected with increasing amounts of DCAF7 expression plasmid, and then infected with the H1N1 (MOI = 0.01) and H3N2 viruses (MOI = 0.02), respectively. The immunoblotting results demonstrated that DCAF7 decreased the levels of NP protein in a dose-dependent manner (Fig. 2D; Fig. S1A). Next, the control and DCAF7-expressing A549 cells were infected with the H1N1 (MOI = 0.1) and H3N2 viruses (MOI = 0.2), expression of DCAF7 significantly reduced NP protein levels of both viruses and viral growth at the indicated infection time points (Fig. 2E; Fig. S1B). To further determine the antiviral effect of DCAF7 on IAV, the control and DCAF7-expressing HEK293T cells were infected with the H1N1 and H3N2 viruses at different multiplicities of infection. The immunoblotting results indicated that DCAF7 also decreased the viral NP protein expression at low MOI = 0.1 or high MOI = 1 (Fig. 2F; Fig. S1C). Finally, immunofluorescence assays were also performed to visualize viral restriction by DCAF7 (Fig. 2G; Fig. S1D). Taken together, these results showed that DCAF7 inhibits IAV replication.

### Knockdown of endogenous DCAF7 promotes influenza A virus replication

**Fig 3:**
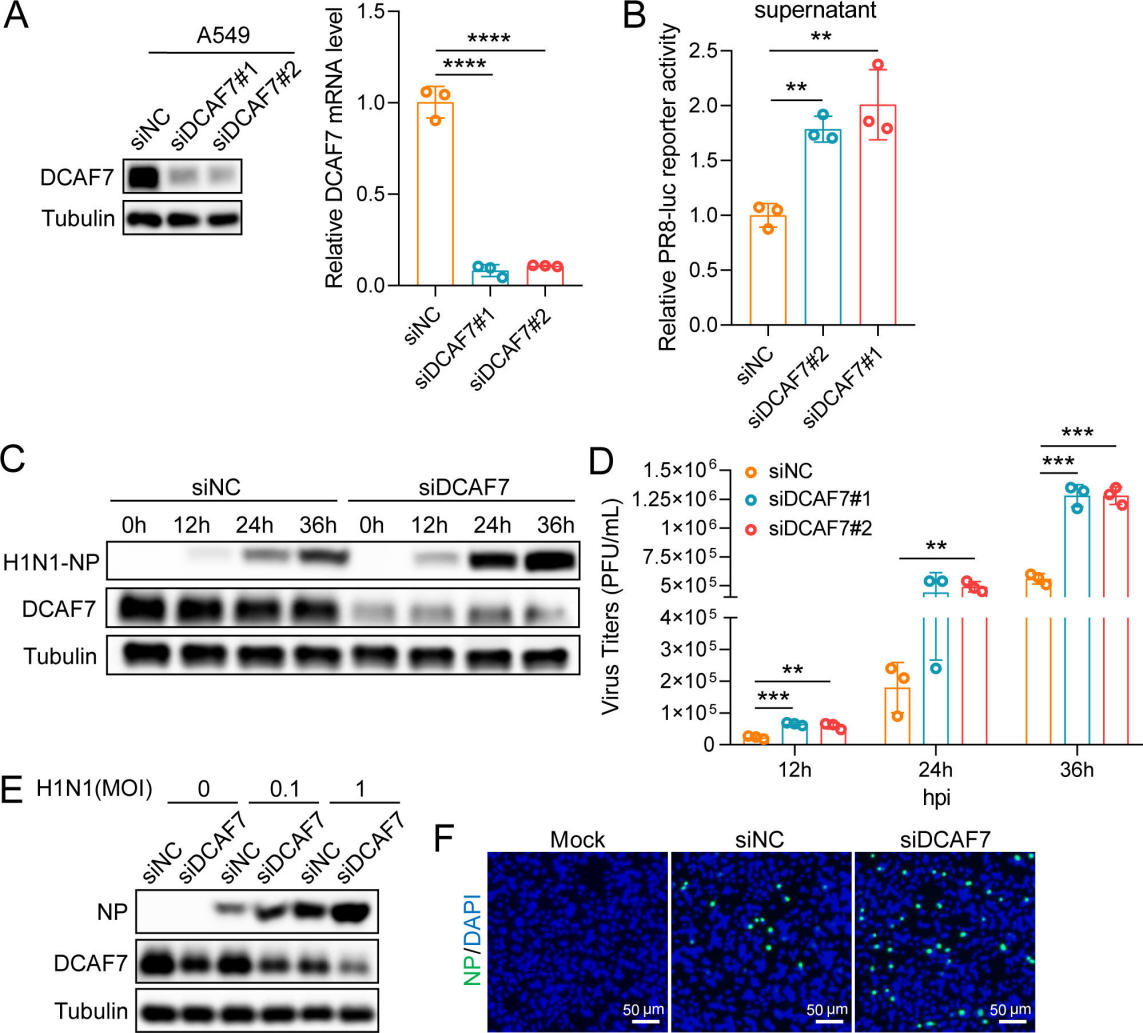
Knockdown of endogenous DCAF7 promotes influenza A virus replication. (A) A549 cells were transfected with siRNAs targeting DCAF7 or negative control (NC) for 48 h, and the knockdown efficiency of DCAF7 was detected by Western blotting (left) and qRT-PCR (right). (B) A549 cells were transfected with siRNAs for 24 h and infected with PR8-Rluc virus for 48 h. The luciferase activity in the supernatants was determined. (C) A549 cells were transfected with siRNAs for 24 h, then infected with H1N1 at an MOI of 0.1, and the viral NP protein level was examined at the indicated time points. (D) A549 cells were transfected with siRNAs for 24 h, then infected with H1N1 at an MOI of 0.1. The virus titers of cell supernatants were determined by plaque assays on MDCK cells. (E) A549 cells were transfected with siRNAs for 24 h and infected with H1N1 (MOI = 0, 0.1, 1) for 24 h. The viral NP protein level was examined by Western blotting. (F) A549 cells were transfected with siRNAs for 24 h, then infected with H1N1 at an MOI of 0.1 for 24 h. An immunofluorescence assay was performed and then visualized by fluorescence microscopy (NP: green; Nuclear: blue), scale bar = 50 µm. An unpaired t-test was used for data statistical analysis, and the data were shown as mean ± SD from three independent experiments. **P < 0.01, ***P < 0.001, ****P < 0.0001.

To further confirm the above results, we knocked down DCAF7 to check the effect on influenza A virus replication using small interfering RNA (siRNA). The knockdown efficiency of endogenous DCAF7 in A549 cells was measured by qRT-PCR and Western blotting analysis (Fig. 3A). A549 cells treated with specific siRNA targeting DCAF7 or control were infected with the PR8-Rluc virus, and then the luciferase activity in the supernatants was measured. The result showed that DCAF7 knockdown led to increased viral reporter activity in A549 cells (Fig. 3B). Silencing DCAF7 increased the NP protein expression and virus titers of H1N1 and H3N2 viruses in A549 cells at the indicated infection time points (Fig. 3C and D; Fig. S2A and B). Similarly, siRNA-treated A549 cells were infected with H1N1 and H3N2 viruses at different multiplicity of infection (MOI = 0, 0.1, 1). The NP protein significantly increased in DCAF7 knockdown A549 cells compared with the control cells (Fig. 3E; Fig. S2C). Furthermore, the replication of IAV was determined by immunofluorescence staining of NP protein-positive cells. As expected, the knockdown of DCAF7 promoted the replication of IAV (Fig. 3F; Fig. S2D).

### Restoration of DCAF7 expression inhibits influenza A virus replication

**Fig 4:**
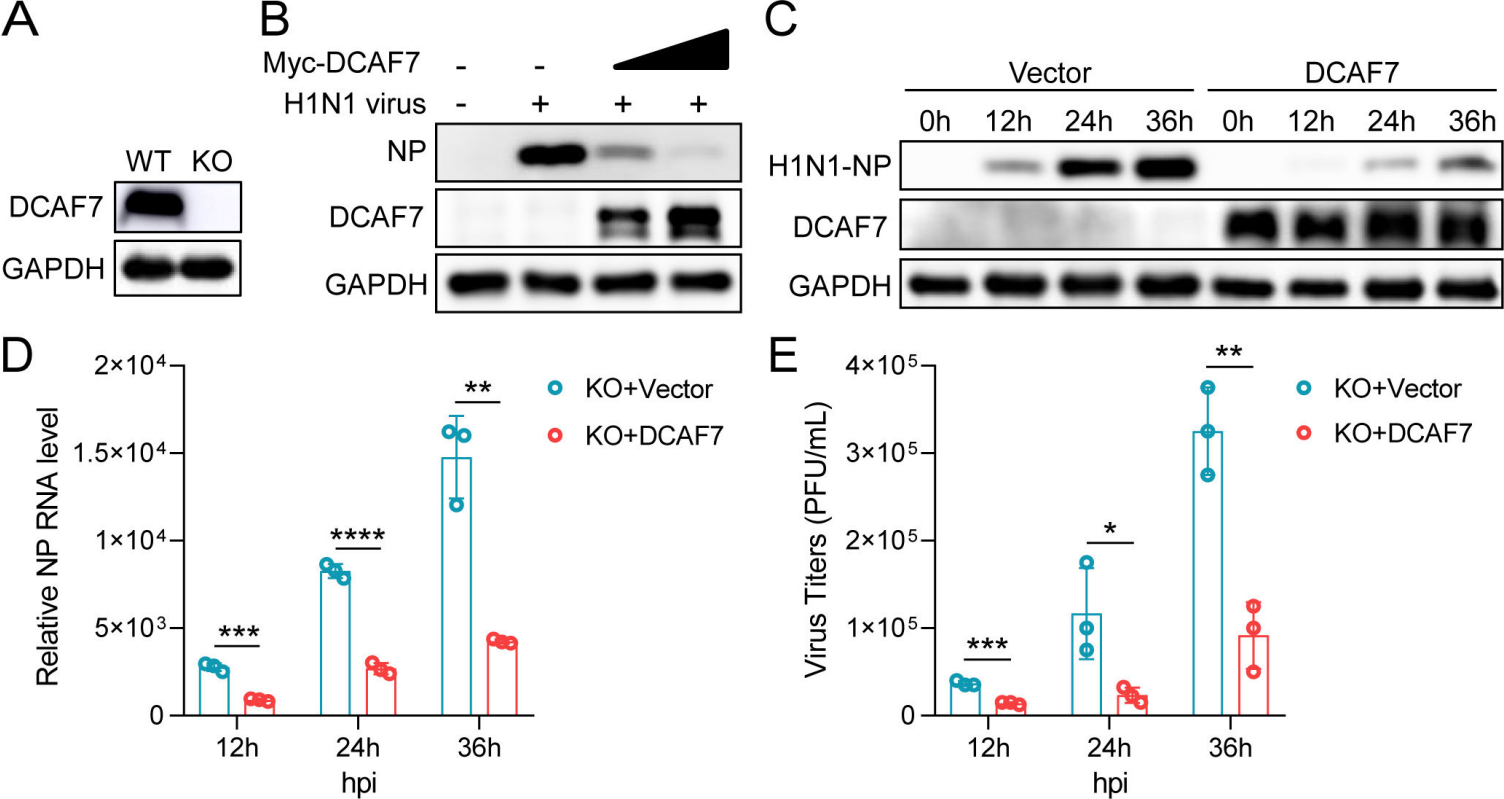
Restoration of DCAF7 expression inhibits influenza A virus replication. (A) The DCAF7-deficient A549 cell line was constructed by the CRISPR/Cas9 system, and the knockout effect of DCAF7 was verified by Western blotting. (B) The DCAF7-deficient A549 cell line was transfected with Myc-tagged DCAF7 protein expression plasmid (0, 0.5, and 1 µg) for 24 h, and the viral NP protein level was examined after 24 h infection with H1N1 viruses at an MOI of 0.1. (C–E) The DCAF7-deficient A549 cell line was transfected with Myc-tagged DCAF7 protein expression plasmid or empty vector control (0.5 µg each) for 24 h, and the viral NP protein (C) and RNA (D) abundance were detected after infection with H1N1 (MOI = 0.1). The virus titers of cell supernatants were determined by plaque assays on MDCK cells (E). An unpaired t-test was used for data statistical analysis, and the data were shown as mean ± SD from three independent experiments. *P < 0.05, **P < 0.01, ***P < 0.001, ****P < 0.0001.

To further verify that the enhancement of IAV replication ability was indeed caused by specific depletion of DCAF7 but not by off-target effects, we detected whether the antiviral effect of DCAF7 on IAV could be rescued by restoring DCAF7 expression in DCAF7 knockout (KO) A549 cells. First, a DCAF7 knockout A549 cell line was generated using the CRISPR/Cas9 system, and the knockout effect of DCAF7 was confirmed by Western blotting with DCAF7 specific antibody (Fig. 4A). Next, the DCAF7 knockout A549 cells were transfected with increasing amounts of DCAF7 expression plasmid. 24 hours post-transfection, cells were infected with H1N1 and H3N2 viruses. The viral NP protein expression was significantly reduced with the increase in DCAF7 expression (Fig. 4B; Fig. S3A). The restoration of DCAF7 expression also reduced viral NP protein and RNA levels in DCAF7 knockout cells compared with the control cells at the indicated infection time points (Fig. 4C and D; Fig. S3B and C). In addition, we found that the virus titers of H1N1 and H3N2 viruses were decreased after the restoration of DCAF7 expression by plaque assays (Fig. 4E; Fig. S3D).

### DCAF7 inhibits influenza A virus polymerase activity and the synthesis of viral RNA, cRNA, and mRNA

**Fig 5:**
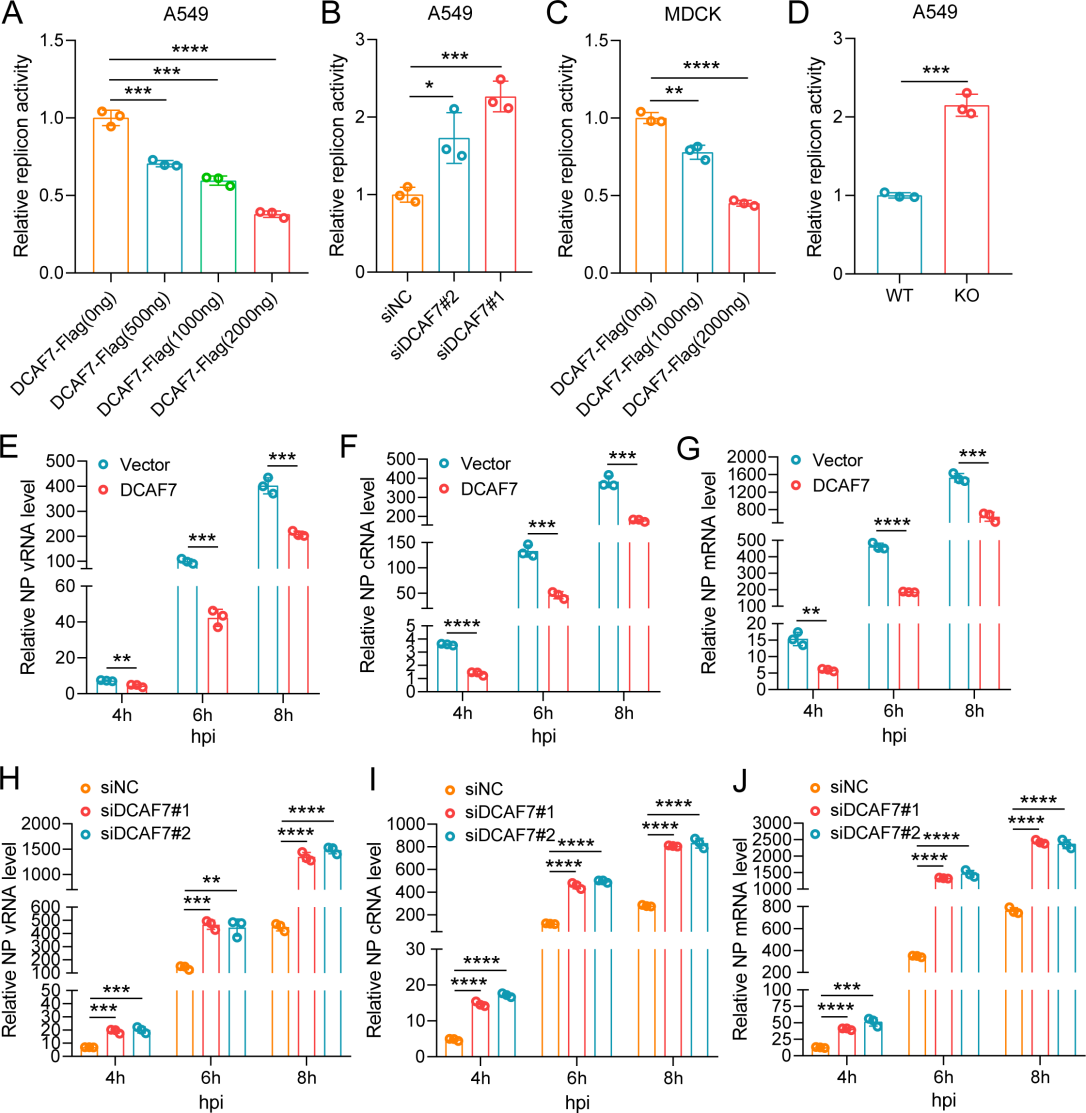
DCAF7 inhibits influenza A virus polymerase activity and the synthesis of viral RNA, cRNA, and mRNA. (A) A549 cells were co-transfected with the Flag-DCAF7 plasmid (0, 0.5, 1, and 2 µg), viral protein expression plasmids (pCAGGS-PB1, pCAGGS-PB2, pCAGGS-PA, and pCAGGS-NP), the luciferase reporter pPolI-NP-Luc and pTK-RL. The luciferase activity was measured after 48 h of transfection. (B) A549 cells were co-transfected with siRNAs targeting DCAF7 or negative control (NC) and the above IAV minireplicon system plasmids for 48 h, and the luciferase activity was measured. (C) MDCK cells were co-transfected with the Flag-DCAF7 plasmid (0, 1, and 2 µg) and the above IAV minireplicon system plasmids for 48 h, and the luciferase activity was measured. (D) DCAF7 KO A549 cells were co-transfected with the IAV minireplicon system plasmids for 24 h, and the luciferase activity was measured. (E–G) A549-DCAF7/A549-Vector cells were infected with H1N1 (MOI = 1) for 4, 6, and 8 h, and then the abundance of NP vRNA (E), cRNA (F), and mRNA (G) was examined by qRT-PCR. (H–J) A549 cells were transfected with siRNAs targeting DCAF7 or negative control (NC) for 36 h, and then infected with H1N1 (MOI = 1) for 4, 6, and 8 h. The abundance of NP vRNA (H), cRNA (I), and mRNA (J) was examined by qRT-PCR. Unpaired t-test was used for data statistical analysis, and the data were shown as mean ± SD from three independent experiments. *P < 0.05, **P < 0.01, ***P < 0.001, ****P < 0.0001.

The above results demonstrated that DCAF7 inhibits IAV infection. To uncover the underlying mechanisms, we first conducted attachment and entry experiments in HEK293T and A549 cells. Cells were incubated with H1N1 (MOI = 1) for 1 h at 4 °C to allow virus binding to the cell surface, followed by incubation with pre-warmed DMEM at 37 °C to allow virus entry. The NP vRNA level of H1N1 was measured by qRT-PCR, and the results showed that DCAF7 did not affect the attachment and entry of IAV (Fig. S4A through D). Next, we explored whether DCAF7 regulates the transcription or replication of IAV and examined the effect of DCAF7 on RdRp activity through minireplicon assay in DCAF7-overexpressing, -knockdown, or -knockout cells. The minireplicon system determines the overall transcriptional and replication activity of the IAV polymerase complex (15, 33). We found that the viral polymerase activity decreased with the increase in DCAF7 expression level in A549 and MDCK cells (Fig. 5A and C). On the contrary, the viral polymerase activity was higher in DCAF7-knockdown and -knockout A549 cells than in the control cells (Fig. 5B and D). To rule out the effect of DCAF7 on luciferase itself, we validated it using the EV-A71 IRES-driven translational system. This system contained an EV-A71 5′ UTR between *Renilla* and *Firefly* luciferase region, and the EV-A71 5′ UTR region was responsible for FLuc translation and expression (34). We observed a similar ratio of FLuc/RLuc in DCAF7-overexpressing HEK293T cells compared with the control cells (Fig. S4E), which suggested DCAF7 has no effect on luciferase itself. Furthermore, we determined whether DCAF7 affects IAV RNA synthesis. DCAF7-overexpressing A549 cells or DCAF7-silenced A549 cells were infected with the H1N1 virus, then the NP vRNA, cRNA, and mRNA abundance were detected at the indicated infection time points by qRT-PCR. As expected, the levels of NP vRNA, cRNA, and mRNA were reduced in DCAF7-overexpressing A549 cells (Fig. 5E through G). However, the levels of the three viral RNAs were significantly increased in the DCAF7-silenced A549 cells compared with those in the control cells (Fig. 5H through J). These results demonstrated that DCAF7 inhibits influenza A virus polymerase activity and the synthesis of viral RNA, cRNA, and mRNA.

### DCAF7 interacts with viral polymerase subunits PB1, PB2, and PA

**Fig 6:**
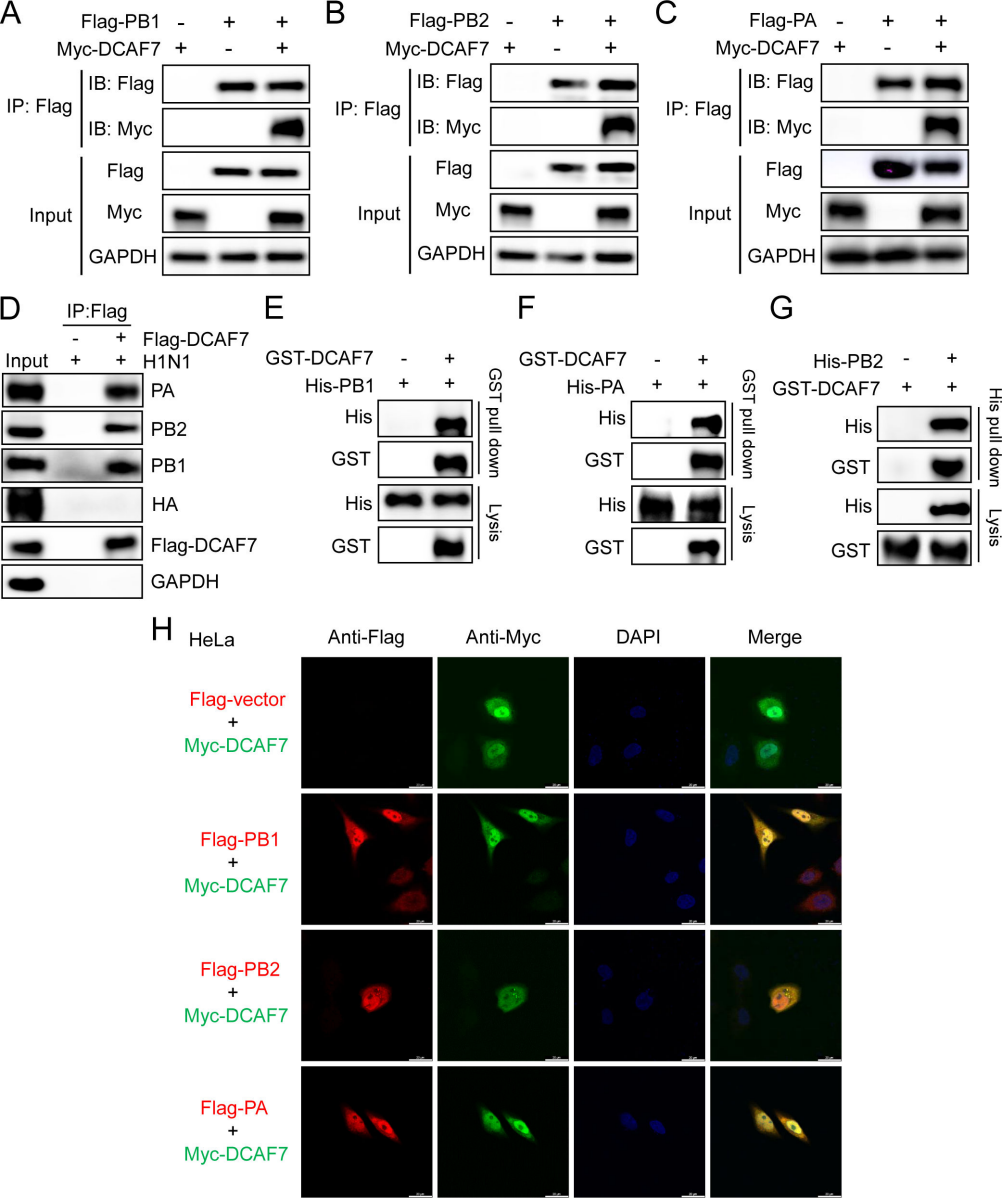
DCAF7 interacts with viral polymerase subunits PB1, PB2, and PA. (A–C) 293T cells were co-transfected with Flag-tagged PB1 (A), PB2 (B), or PA (C) expression plasmid and Myc-tagged DCAF7 expression plasmid or empty vector controls for 48 h, then co-immunoprecipitation (Co-IP) assay was performed with anti-Flag antibody. (D) Interactions between DCAF7 and viral polymerase subunits PB1, PB2, and PA during IAV infection. 293T-DCAF7/293T-Vector cells were infected with H1N1 (MOI = 1) for 24 h, and then immunoprecipitated with anti-Flag antibody. The immunocomplexes were analyzed by Western blotting with the indicated antibodies. Influenza A virus hemagglutinin (HA) protein was used as a negative control. (E–G) Pull-down assays of DCAF7 and polymerase subunits PB1/PA/PB2. GST-DCAF7 and His-PB1/PA/PB2 proteins were expressed in BL21 cells and purified. The purified GST-DCAF7 and His-PB1 (E), His-PA (F), or His-PB2 (G) proteins were incubated together for pull-down. (H) Colocalization of DCAF7 and viral polymerase subunits in HeLa cells. HeLa cells were co-transfected with Myc-DCAF7 and Flag-PB1/PB2/PA or empty vector control for 36 h. The cells were fixed and incubated with anti-Flag mouse antibody and anti-Myc rabbit antibody, followed by incubation with goat anti-rabbit IgG conjugated with Alexa Fluor 488 (green), goat anti-mouse IgG conjugated with Alexa Fluor 555 (red). The cell nuclei were stained with DAPI (blue), and images were captured using a confocal microscope. Scale bar = 20 µm.

As DCAF7 inhibits viral polymerase activity and viral RNA synthesis, and previous studies showed DCAF7 may be associated with the viral polymerase. Therefore, we hypothesized that DCAF7 may inhibit IAV through targeting the viral polymerase complex. To test this hypothesis, Flag-PB1, Flag-PB2, or Flag-PA and Myc-DCAF7 plasmids were co-transfected into HEK293T cells, and then co-immunoprecipitation (Co-IP) was performed with anti-Flag antibody. The results showed that DCAF7 interacts with PB1, PB2, and PA (Fig. 6A through C). We then determined whether DCAF7 interacts with the polymerase subunits under IAV infection. Flag-DCAF7 overexpressing HEK293T cells were infected with H1N1 for 24 h, and then cell lysates were immunoprecipitated with anti-Flag antibody. We found that DCAF7 interacts with the polymerase subunits during IAV infection (Fig. 6D). Furthermore, the pull-down experiments showed that DCAF7 can directly interact with viral polymerase subunits (Fig. 6E through G). To detect the colocalization between DCAF7 and viral PB1, PB2, or PA, HeLa cells were co-transfected with Myc-DCAF7 and Flag-PB1, Flag-PB2, or Flag-PA plasmids to perform an indirect immunofluorescence assay. We found that DCAF7 and the viral polymerase subunits colocalized in the cytoplasm and nucleus (Fig. 6H).

The above results prove that DCAF7 interacts with IAV polymerase subunits. To identify the key region of polymerase subunits responsible for the interaction with DCAF7, we constructed a series of the truncation mutants of viral polymerase subunits based on previous studies (Fig. S5A, C and E) (18, 32, 35, 36). Myc-DCAF7 and Flag-PB1 or truncation mutants were co-transfected into HEK293T cells for 48 h, and co-immunoprecipitation was performed with anti-Flag antibody. The results showed that the N-terminal (1 to 265 amino acid residues) of PB1 mainly mediated the interaction with DCAF7 (Fig. S5B). Similarly, both the N terminus and C terminus of PB2 can interact with DCAF7 (Fig. S5D); while the C-terminal (252 to 716 amino acid residues) of PA binds to DCAF7 (Fig. S5F).

### DCAF7 mediates K48-linked polyubiquitination of PA at the K609 site and its degradation

**Fig 7:**
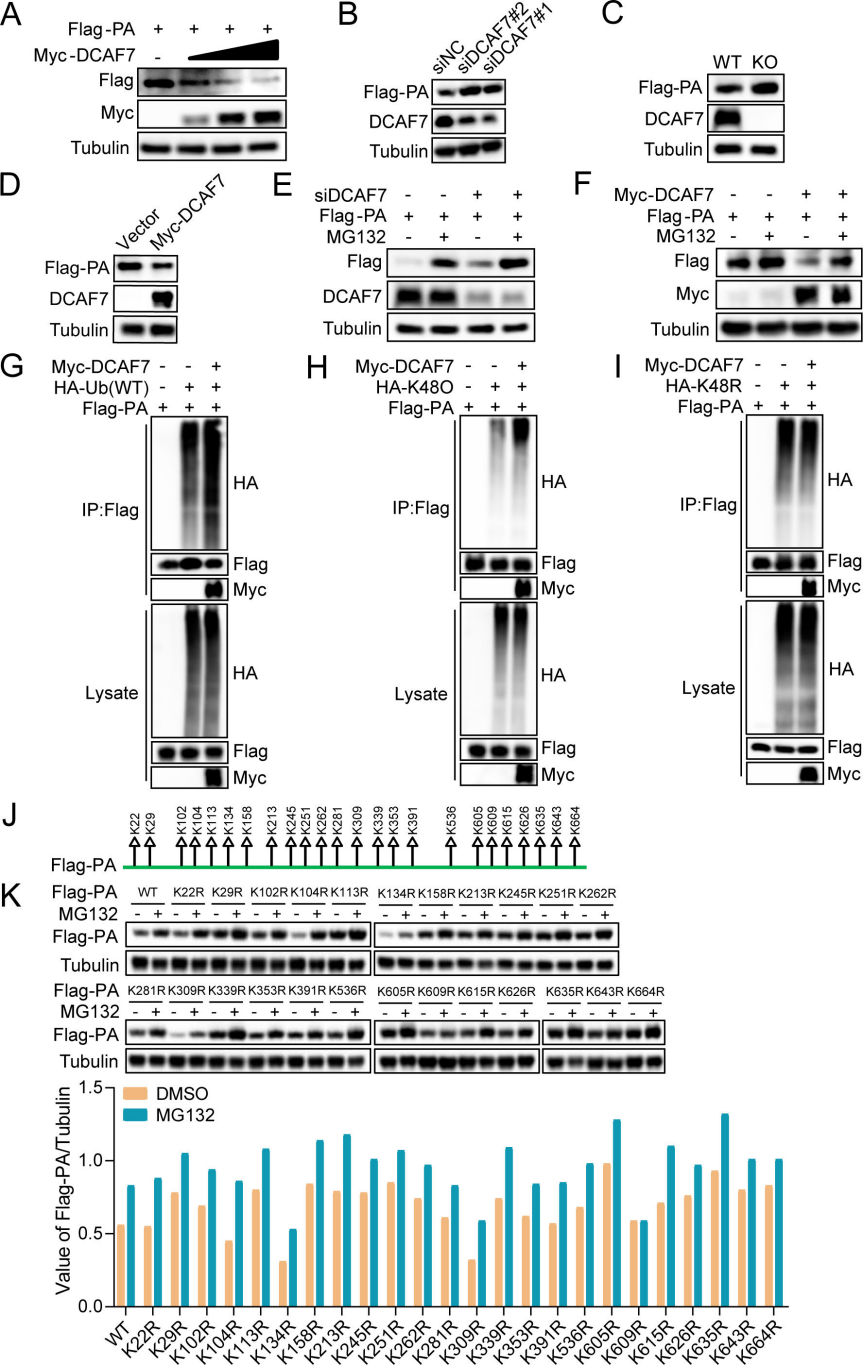
DCAF7 mediates K48-linked polyubiquitination of PA at the K609 site and its degradation. (A) HEK293T cells were co-transfected with Myc-tagged DCAF7 plasmid (0, 0.5, 1, and 2 µg) and Flag-tagged PA plasmid (1 µg) for 48 h, and the expression of PA and DCAF7 was examined by Western blotting with the indicated antibodies. (B) HEK293T cells were co-transfected with siRNAs targeting DCAF7 or negative control (NC), Flag-tagged PA plasmid (1 µg) for 36 h. The expression of PA and DCAF7 was examined by Western blotting. (C) The WT and DCAF7 KO A549 cells were transfected with Flag-tagged PA plasmid for 36 h, and the expression of PA was examined by Western blotting. (D) The DCAF7-deficient A549 cells were transfected with Myc-tagged DCAF7 or empty vector and Flag-tagged PA plasmid for 36 h, and the expression of PA and DCAF7 was examined by Western blotting. (E) HEK293T cells were transfected with siRNA targeting DCAF7 or negative control (NC) and Flag-tagged PA plasmid for 36 h, and then the cells were treated with or without MG132 (20 µM) for 12 h. The cells were lysed, and cell lysates were detected. (F) The DCAF7-deficient A549 cells were transfected with Myc-tagged DCAF7 or empty vector and Flag-tagged PA plasmid for 36 h. Then, the cells were treated with or without MG132 (20 µM) for 12 h and were analyzed by Western blotting. (G–I) HEK293T cells were co-transfected with HA-Ub (WT) (G), HA-K48O (H), HA-K48R (I), Myc-DCAF7, and Flag-PA plasmids for 48 h, and then immunoprecipitation assays were performed with anti-Flag antibody. WT, wild type; KO, K only; KR, K is mutated to R. (J) Ubiquitin-modified lysine sites in the IAV polymerase PA subunit. (K) HEK293T cells were transfected with PA-WT and all PA mutant plasmids for 36 h and treated with MG132 (10 µM) for 12 h, and the protein abundance of PA was determined by Western blotting and quantified with software.

The above results indicated that DCAF7 interacts with the subunits of IAV polymerase, and this interaction may be responsible for the inhibition of the polymerase activity. Previous studies have shown that DCAF7 interacts with target proteins and degrades them via the ubiquitin-proteasome pathway (22, 25); therefore, we hypothesized that DCAF7 may reduce polymerase activity by degrading viral polymerase subunits. To test this hypothesis, HEK293T cells were co-transfected with increasing amounts of Myc-DCAF7 plasmid and a constant amount of Flag-PB1, PB2, or PA plasmids. The immunoblotting results revealed that DCAF7 reduced the expression of viral PA in a dose-dependent manner (Fig. 7A), but did not affect the expression of PB1 and PB2 (Fig. S6A and B). Moreover, DCAF7 did not affect the mRNA level of viral PA (Fig. S6E). Consistently, the knockdown of DCAF7 increased the viral PA protein expression in HEK293T cells (Fig. 7B), but also did not affect PB1 and PB2 (Fig. S6C and D). Moreover, the knockout of DCAF7 enhanced the expression of PA (Fig. 7C), and restoration of DCAF7 expression in DCAF7-knockout A549 cells decreased the expression of viral PA (Fig. 7D). These results suggested that DCAF7 specifically reduces the stability of PA protein. Since the autophagy-lysosome and ubiquitin-proteasome pathways are the main protein degradation pathways in eukaryotic cells, we suspected that DCAF7 may weaken the stability of PA protein through these protein degradation pathways. Then HEK293T cells were transfected with Flag-tagged PA plasmid and specific siRNA targeting DCAF7 or control for 36 h. The cells were treated with proteasome inhibitor MG132, autophagy inhibitor 3MA, or lysosomal inhibitor CQ for 12 h, respectively. The immunoblotting results revealed that the proteasome inhibitor MG132 significantly rescued the degradation of PA protein (Fig. 7E), while lysosomal inhibitor CQ and autophagy inhibitor 3MA had no effect on it (Fig. S6F and G). By contrast, DCAF7-knockout A549 cells were transfected with Flag-tagged PA and Myc-tagged DCAF7 plasmids for 36 h and treated with MG132 for 12 h. We found that the expression of DCAF7 decreased the PA protein abundance (Fig. 7F, lane 3), which is rescued by treatment with MG132 (Fig. 7F, lane 4). Taken together, these results suggest that DCAF7 reduces the expression level of PA protein through the ubiquitin-proteasome degradation pathway.

Furthermore, the ubiquitination assay showed that the polyubiquitination level of PA was enhanced by DCAF7 expression (Fig. 7G). There are seven lysine residues within ubiquitin that allow site-specific ubiquitination to occur at K6, K11, K27, K29, K33, K48, or K63 sites. We further identified the types of polyubiquitin chains of PA protein that were mediated by DCAF7. HEK293T cells were co-transfected with Myc-DCAF7, Flag-PA, or ubiquitin plasmids that contain only a single lysine residue to perform ubiquitination assays. The results indicated that the expression of DCAF7 promoted the K48-linked ubiquitination of PA (Fig. 7H), but not K6, K11, K27, K29, K33, or K63-linked ubiquitination of PA (Fig. S7A through F). Consistently, we found that DCAF7 failed to increase the polyubiquitination of PA when K48 was mutated to arginine (Fig. 7I). These results suggest that DCAF7 promoted the K48-linked ubiquitination of PA and its degradation. Two previous studies revealed the potential ubiquitin modification sites of influenza A virus PA protein by mass spectrometry (37, 38). To identify the ubiquitination sites in the PA subunit mediated by DCAF7, a total of 24 ubiquitin-modified lysine sites in PA protein were mutated by arginine substitution (Fig. 7J). We detected the stability of PA mutants with or without MG132 treatment and found that only the K609R was not affected (Fig. 7K), and this result indicated that the K609 site was required for the ubiquitin degradation of PA protein. Next, we examined the ubiquitination of PA-K609R protein in HEK293T cells transfected with Myc-DCAF7 and found that the mutation of K609R greatly weakened PA ubiquitination with the expression of DCAF7, while the ubiquitination of PA-K22R (negative control) was not significantly changed compared with PA-WT (Fig. S7G). Thus, the K609 site is the main target for ubiquitination of PA by DCAF7.

### CRL4B^DCAF7^ E3 ligase targets viral polymerase subunit PA for degradation

**Fig 8:**
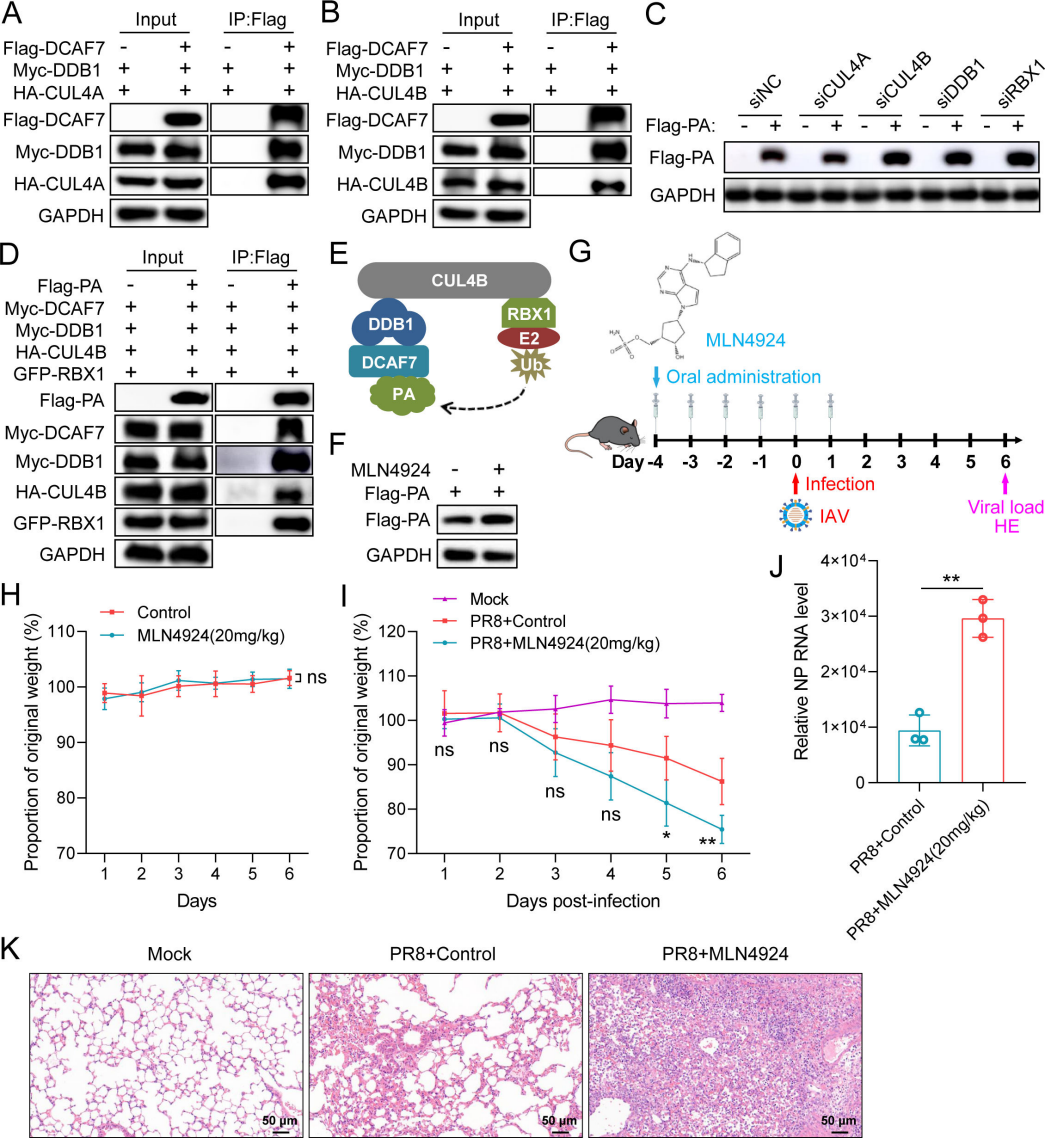
CRL4B^DCAF7^ E3 ligase targets viral polymerase subunit PA for degradation. (A and B) HEK293T cells were transfected with indicated plasmids for 48 h, then the cells were lysed and immunoprecipitated with anti-Flag antibody for immunoblotting analysis. (C) CUL4A, CUL4B, DDB1, and RBX1 were knocked down separately in HEK293T cells by siRNAs, and the knockdown cells were transfected with Flag-PA or empty vector for 48 h. The expression of PA was analyzed by Western blotting. (D) PA interacts with DCAF7, CUL4B, DDB1, and RBX1. HEK293T cells were co-transfected with Myc-DCAF7, Myc-DDB1, HA-CUL4B, GFP-RBX1, Flag-PA expression plasmids or empty vector for 48 h, and then co-immunoprecipitation (Co-IP) assay was performed with anti-Flag antibody. (E) Schematic of CRL4B^DCAF7^ mediated PA degradation. Scaffold protein CUL4B forms a complete E3 ligase complex with the RING-box protein RBX1, the adaptor protein DDB1, and the substrate recognition receptor DCAF7. (F) HEK293T cells were transfected with Flag-tagged PA expression plasmid for 36 h, the cells were treated with or without MLN4924 (50 µM) for 12 h and were analyzed by Western blotting. (G) Schematic diagram of MLN4924 inhibitor treatment and PR8 infection mouse model. (H) Toxicity evaluation of MLN4924 inhibitor in mice. C57BL/6J mice were treated with MLN4924 (20 mg/kg) for 6 days and body weight was monitored. (I–K) C57BL/6J mice were treated with MLN4924 (20 mg/kg) for 6 days and then infected with mouse-adapted PR8 (417 PFU) to observe the body weight changes (I), measure the viral load of lung tissues (J), and evaluate the pathological characteristics (K). An unpaired t-test was used for data statistical analysis, and the data were shown as mean ± SD from three independent experiments. ns, no significance, *P < 0.05, **P < 0.01.

The above data suggest that DCAF7 mediates PA degradation by enhancing K48-linked ubiquitination. Next, we explored how DCAF7 degrades PA protein. Cullin4-RING (CRL4) E3 ligase is a multiprotein complex that specifically recognizes substrates for ubiquitin degradation. The CRL4 E3 ligase is composed of Cullin4 (CUL4A/CUL4B), DNA damage-binding protein-1 (DDB1), and RING-box protein 1 (RBX1). CUL4A or CUL4B serves as a scaffold protein to recruit DDB1 and RBX1 to assemble the DDB1-CUL4-RBX1 complex. RBX1 as a RING finger protein binds the E2 ubiquitin-conjugating enzyme, and DDB1 acting as an adapter recruits DDB1- and CUL4-associated factors (DCAFs) to form complete CUL4-RING E3 ubiquitin ligases (39). DCAFs are responsible for substrate recognition and determining substrate specificity in the CUL4-RING E3 ubiquitin ligases (40). DCAF7 (DDB1 and CUL4-associated factor 7) is a novel protein identified in DDB1 complexes and inferred to function as a substrate receptor in the CRL4 E3 ubiquitin ligase complexes (20). To investigate whether DCAF7 acts as a substrate recognition receptor to form a complete CUL4-RING E3 ligase-degrading IAV polymerase PA protein with the RBX1-CUL4-DDB1 complexes. We first examined the interaction between DCAF7 and DDB1, CUL4A, or CUL4B and found that DCAF7 associates with all of them (Fig. 8A and B). Subsequently, we used siRNA to identify whether CUL4A or CUL4B plays a role in PA degradation. The results validated that CUL4B was responsible for PA degradation, and CUL4A knockdown had no impact on it (Fig. 8C, lanes 4 and 6; Fig. S8A). Besides, the knockdown of DDB1 and RBX1 also enhanced the stability of PA (Fig. 8C, lanes 8 and 10; Fig. S8A). These data suggest that the DDB1-CUL4B-RBX1 complex was involved in regulating the stability of PA. However, the DDB1-CUL4-RBX1 complex had no obvious impact on the stability of PB1 and PB2 (Fig. S8B). Previous results confirmed that DCAF7 physically interacts with PA protein, and DCAF7 reduced the stability of PA. In addition to the DDB1-CUL4B-RBX1 complex, the formation of the complete CUL4B-RING E3 ligases also needs substrate recognition receptors (DCAFs) (39). To further confirm the DCAF7 and DDB1-CUL4B-RBX1 complex formed complete CUL4B-RING E3 ligases targeting PA for degradation, the Flag-PA, Myc-DCAF7, Myc-DDB1, HA-CUL4B, and GFP-RBX1 plasmids were co-transfected into HEK293T cells for co-immunoprecipitation assay. We found that each of these proteins was present in precipitates with anti-Flag antibody (Fig. 8D). To sum up, these results verified that DCAF7, CUL4B, DDB1, and RBX1 form a CRL4B^DCAF7^ E3 ligase and are responsible for PA degradation (Fig. 8E).

To further confirm that CUL4B-RING E3 ligases regulate the expression of PA protein, we employed a specific inhibitor of cullin-RING E3 ligases, MLN4924. Treatment of MLN4924 upregulated the protein level of the IAV-PA subunit and viral polymerase activity (Fig. 8F; Fig. S8C and D). In addition, we also verify the effect of the MLN4924 inhibitor on influenza A virus replication *in vivo*. C57BL/6J mice were treated with 20 mg/kg MLN4924 six times and infected with mouse-adapted PR8 virus (Fig. 8G). Compared to the control group, the weight loss of mice treated with 20 mg/kg MLN4924 for 6 days was not observed, indicating that MLN4924 had no obvious drug toxicity *in vivo* (Fig. 8H). Mice receiving the MLN4924 inhibitor exhibited more weight loss upon viral infection (Fig. 8I). Furthermore, the viral NP RNA abundance in the lung tissues of MLN4924-treated mice was significantly higher than that in the control group (Fig. 8J). The HE staining results of lung tissues from mice on the sixth day after infection also confirmed that mice treated with the MLN4924 inhibitor had more severe pathological changes than those of the untreated group, with more cell and protein debris infiltrating into alveoli and obvious alveolar necrosis (Fig. 8K). These results clearly showed that MLN4924 treatment promoted the replication of IAV *in vivo*, causing more severe damage to the mice.

### Activation of CUL4B by etoposide promotes PA degradation and inhibits the IAV replication

**Fig 9:**
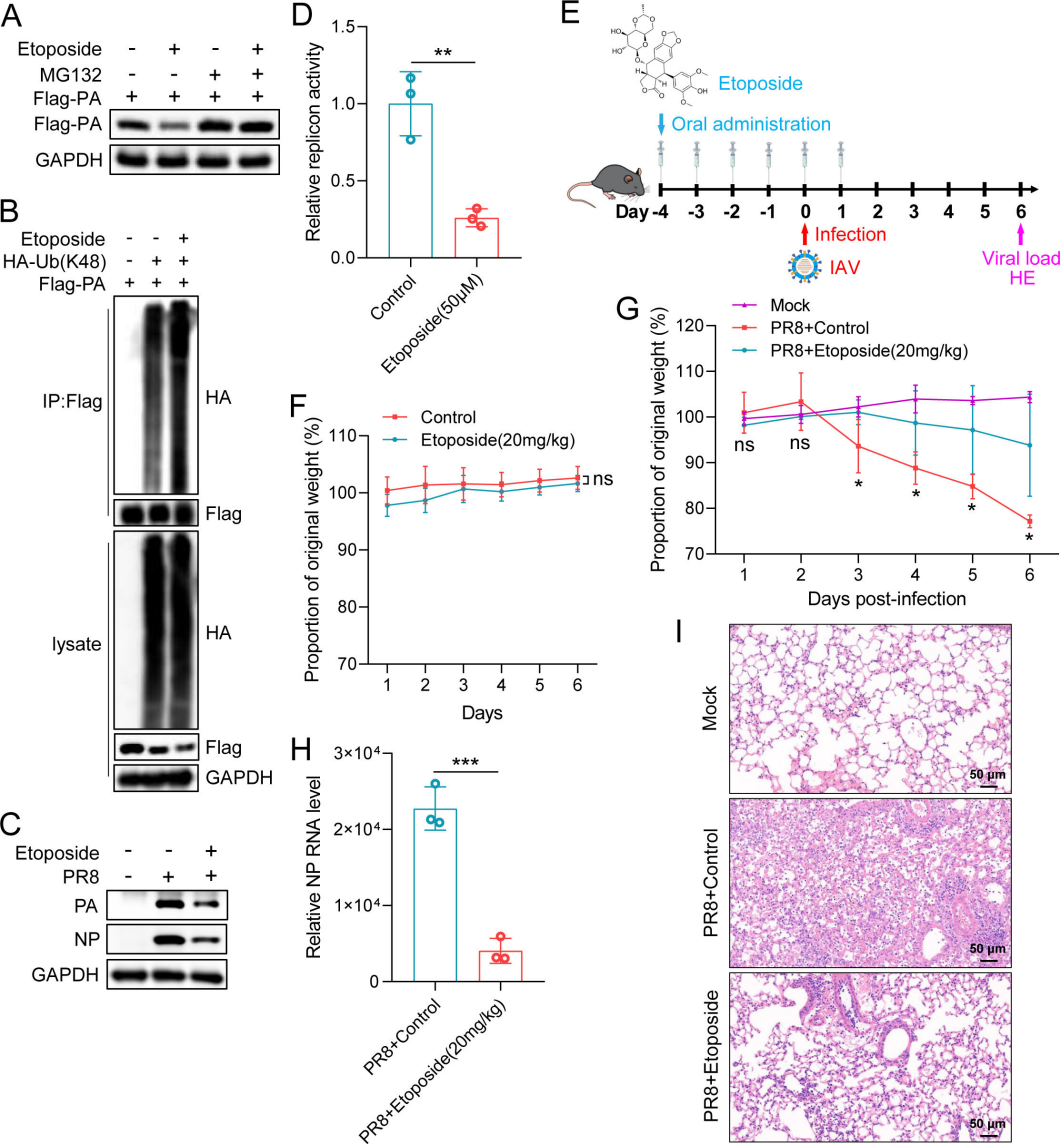
Activation of CUL4B by etoposide promotes PA degradation and inhibits IAV replication. (A) HEK293T cells were transfected with Flag-PA plasmid for 36 h and treated with MG132 (10 µM) or etoposide (50 µM) for 12 h for immunoblotting analysis. (B) HEK293T cells were transfected with the indicated plasmids for 36 h and then treated with etoposide (50 µM) for 12 h. The cells were lysed, and the lysates were subjected to immunoprecipitation (IP) assays with anti-Flag antibody. (C) A549 cells were infected with H1N1 viruses (MOI = 0.1) and treated with etoposide (50 µM) for 24 h to detect viral replication by immunoblotting with NP and PA antibodies. (D) HEK293T cells were transfected with IAV minireplicon system plasmids for 36 h and treated with etoposide (50 µM) for 12 h, and the luciferase activity was measured. (E) Schematic diagram of etoposide drug treatment and PR8 infection mouse model. (F) Toxicity evaluation of etoposide in mice. C57BL/6J mice were treated with etoposide (20 mg/kg) for 6 days and body weight was monitored. (G–I) C57BL/6J mice were treated with etoposide (20 mg/kg) for 6 days and then infected with mouse-adapted PR8 (835 PFU) to observe the body weight changes (G), measure the viral load of lung tissues (H), and evaluate the pathological characteristics (I). An unpaired t-test was used for data statistical analysis, and the data were shown as mean ± SD from three independent experiments. ns, no significance, *P < 0.05, **P < 0.01, ***P < 0.001.

The activity of cullins is regulated by neddylation (41). Neddylation of cullins is necessary to activate cullin-RING ubiquitin ligases, and inhibition of cullins neddylation can inactivate cullin-RING ubiquitin ligases (42, 43). Etoposide is an important anti-tumor drug widely used in the treatment of a variety of human cancers, including small-cell lung cancer, germ-line malignancies, sarcomas, leukemias, and lymphomas (44). Studies have shown that etoposide activates the neddylation of CUL4B and promotes the proteasomal degradation of target proteins (45). However, whether etoposide can promote the degradation of the IAV PA subunit has not been reported. To test this hypothesis, we treated flag-PA-transfected HEK293T cells with etoposide for 12 h for immunoblotting analysis. We found that etoposide treatment increased the degradation of PA protein, while MG132 disrupted the etoposide function (Fig. 9A). Moreover, etoposide increased the K48-linked ubiquitination of PA protein (Fig. 9B). To further explore the antiviral ability of etoposide in IAV infection, A549 cells were infected with PR8 virus and treated with etoposide. It was found that etoposide could weaken the replication of the influenza virus (Fig. 9C). Moreover, etoposide significantly restricted the viral polymerase activity (Fig. 9D). Finally, we also investigated the antiviral effects of etoposide *in vivo*; C57BL/6J mice were treated with 20 mg/kg etoposide six times and infected with mouse-adapted PR8 virus (Fig. 9E). Mice treated with etoposide (20 mg/kg) orally for 6 days did not lose weight, indicating etoposide had no obvious drug toxicity to mice (Fig. 9F). Mice receiving the etoposide treatment exhibited less weight loss than the control group upon viral infection (Fig. 9G). Furthermore, the viral NP RNA abundance in the lung tissues of etoposide-treated mice was significantly lower than that in the control group (Fig. 9H). The HE staining results of lung tissues from mice on the sixth day after infection also confirmed that mice untreated with etoposide drug had more severe pathological changes than the treated group (Fig. 9I).

### Influenza A virus infection downregulates DCAF7 expression *in vitro* and *in vivo*

**Fig 10:**
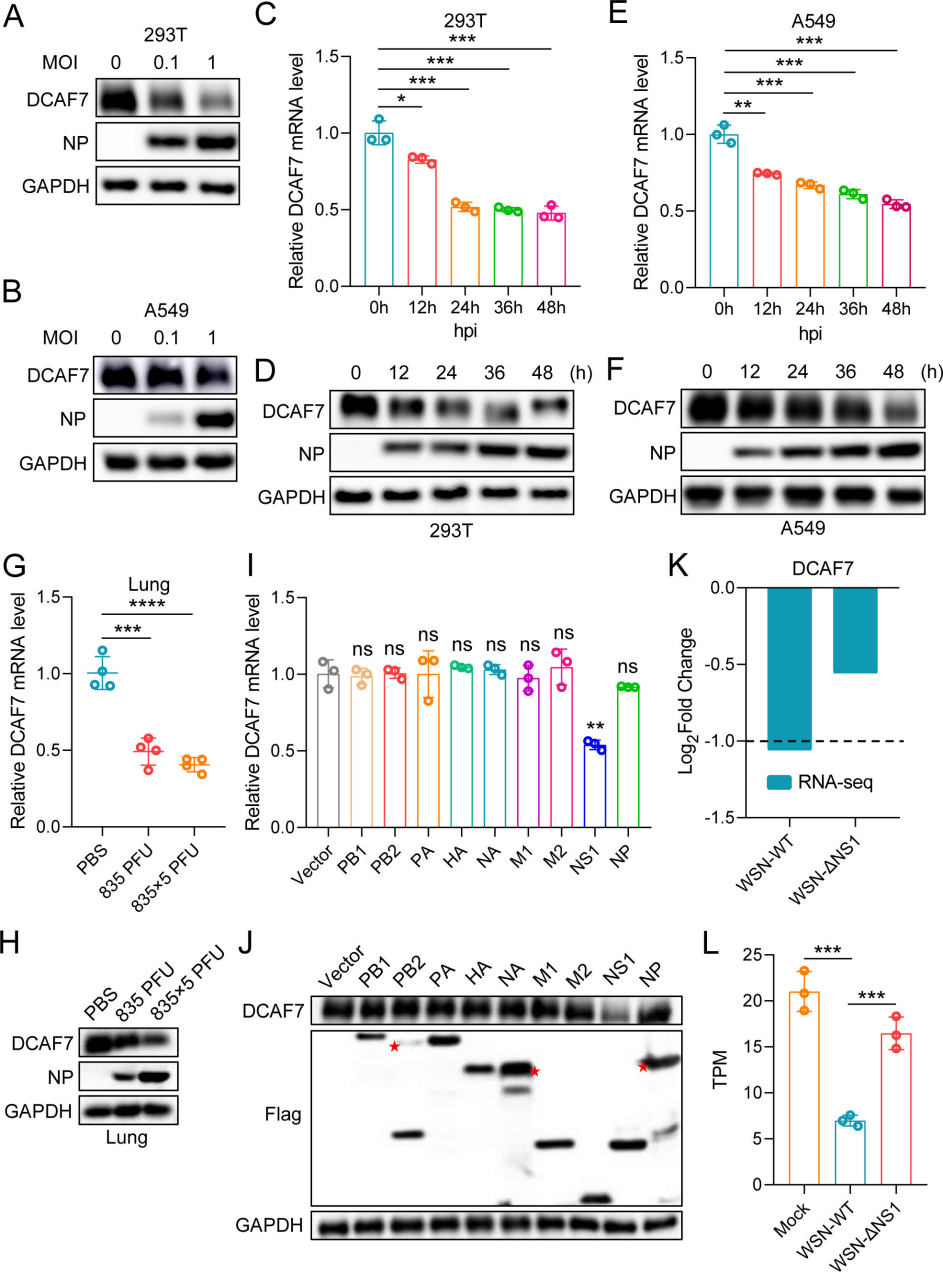
Influenza A virus infection downregulates DCAF7 expression. (A and B) IAV infection reduced DCAF7 expression in a viral dose-dependent manner *in vitro*. HEK293T cells (A) or A549 cells (B) were infected with the H1N1 virus at the indicated MOI for 24 h, and the DCAF7 protein expression was examined. (C and D) HEK293T cells were infected with the H1N1 virus (MOI = 1) at the indicated time points, the mRNA level of DCAF7 was detected by qRT-PCR (C), and the protein level of DCAF7 was measured by Western blotting (D). (E and F) A549 cells were infected with the H1N1 virus (MOI = 1) at the indicated time points, and the mRNA level (E) or protein level (F) of DCAF7 was measured. (G and H) IAV infection reduced DCAF7 expression in a viral dose-dependent manner *in vivo*. Eight-week mice were inoculated with mouse-adapted PR8 virus (0, 835, 835 × 5 PFU), DCAF7 mRNA (G), and protein (H) expression levels were detected in lung tissues 6 days after infection. (I and J) Influenza A virus NS1 triggers downregulation of DCAF7. The IAV-PB1/PB2/PA/HA/NA/M1/M2/NS1/NP protein expression plasmids with Flag tag (1.5 µg each) were transfected into HEK293T cells, and the mRNA (I) and protein (J) levels of endogenous DCAF7 were detected 48 h after transfection. (K and L) RNA-seq data (GSE156152) derived from WSN-WT- and WSN-ΔNS1-infected HEK293T cells were used to analyze the expression changes of DCAF7. The value of Log2FoldChange was calculated by bioinformatics methods (K),︱Log2FoldChange︱>1 represents differential genes. The specific amounts of DCAF7 expression in Mock, WSN-WT, and WSN-ΔNS1 groups (L). TPM: transcripts per million. An unpaired t-test was used for data statistical analysis, and the data were shown as mean ± SD from three independent experiments. ns, no significance, *P < 0.05, **P < 0.01, ***P < 0.001, ****P < 0.0001.

The above studies confirmed that DCAF7 is an intrinsic restriction factor for influenza A virus, which restricts viral replication by degrading viral PA through the ubiquitin-proteasome pathway. Whether the influenza A virus attenuates its antiviral ability by regulating the expression of DCAF7 during infection. To determine DCAF7 expression changes during IAV infection, HEK293T and A549 cells were infected with the H1N1 virus (MOI = 0, 0.1, 1) for 24 h. The immunoblotting results showed that the DCAF7 expression was reduced in both HEK293T and A549 cells (Fig. 10A and B). Subsequently, the mRNA and protein levels of DCAF7 were detected at different time points post-infection. We observed the mRNA level of DCAF7 decreased by half at 48 h.p.i (Fig. 10C and E), and the DCAF7 protein level also decreased significantly after IAV infection (Fig. 10D and F). These data confirmed that DCAF7 expression was reduced during IAV infection *in vitro*. To investigate whether IAV infection also leads to the downregulation of DCAF7 expression *in vivo*, we conducted mouse-adapted PR8 virus infection experiments in mice with different viral doses. These results suggested that IAV infection downregulated the mRNA and protein level of DCAF7 in the lung tissue of mice at 6 d.p.i (Fig. 10G and H). Collectively, these findings confirm that IAV infection downregulates DCAF7 expression *in vitro* and *in vivo*.

Next, we explore the potential mechanism of DCAF7 downregulation induced by IAV infection. Since IAV is inherently sensitive to the antiviral effects of interferon (IFN), we speculate that type I interferon probably regulates DCAF7 expression. Hence, A549 cells were treated with IFN-α or IFN-β, and the mRNA and protein levels of DCAF7 were determined. The Western blotting analysis and qRT-PCR results showed that type I interferon did not regulate DCAF7 expression (Fig. S9A through D). Numerous studies have shown that the influenza A virus can directly regulate the expression of host proteins through viral proteins (46–48), so we suspect that IAV viral proteins also directly regulate the expression of DCAF7. Flag-tagged IAV PB1, PB2, PA, HA, NA, M1, M2, NS1, and NP expression plasmids were transfected into HEK293T cells, respectively, and the mRNA level and protein expression of endogenous DCAF7 were then measured. We observed that only IAV NS1 protein, but not other viral proteins, decreased the mRNA and protein levels of DCAF7 (Fig. 10I and J). Consistently, Chen et al. infected HEK293T cells with wild-type influenza WSN or with a mutant virus deficient in encoding fully functional NS1. These infection experiments were used to perform RNA-Seq analysis to evaluate significant changes in the transcriptome of infected cells (GSE156152). We found that the mRNA expression of DCAF7 was reduced in WSN-infected cells, and NS1-deficient WSN could not significantly reduce DCAF7 expression compared to non-infected cells from the above RNA sequencing results (Fig. 10K and L). At present, the DCAF (DDB1 and CUL4-associated factor) family contains dozens of members (40), and we tested some major members of this family to determine whether IAV NS1 can also downregulate their expression. qRT-PCR results showed that viral NS1 did not affect the expression of DCAF2, DCAF3, DCAF5, DCAF6, DCAF9, DCAF11, DCAF13, and DCAF14 (Fig. S9E through L). The results suggest that the downregulation of DCAF7 expression by NS1 may be relatively specific. Taken together, these findings suggest that DCAF7 expression is decreased during IAV infection and is caused by the viral NS1 protein.

## DISCUSSION

IAV is an important human viral pathogen that causes severe respiratory infections. Influenza, also known as the flu, is an acute respiratory illness that occurs all over the world and seriously affects human health and the social economy. Vaccination is an effective measure to prevent the occurrence and epidemic of viral diseases. At present, inactivated vaccines, live attenuated vaccines, and recombinant vaccines are recommended for influenza prevention (49). However, owing to the rapid antigenic variation of the influenza virus, numerous viral subtypes, and individual differences in immune response, vaccines cannot achieve a complete prevention effect during epidemics. Antiviral drugs are also widely used to treat influenza, and four antiviral drugs for the treatment of influenza have been approved, including neuraminidase (NA) inhibitors (oseltamivir, zanamivir, and peramivir) and PA inhibitor (baloxavir) (50). However, antiviral drugs gradually lose their efficacy due to the emergence of drug-resistant strains. For example, oseltamivir-resistant H1N1 strains began to emerge during the 2007–2008 influenza season (51). Therefore, it is urgent to find new therapeutic approaches for IAV. The IAV polymerase is conserved among different viral subtypes, and its activity is essential for viral replication and propagation. Identifying the key host factors that influence IAV polymerase can provide new anti-influenza drug development strategies. In this work, we identified DCAF7 as an IAV antiviral factor that significantly inhibited the proliferation of H1N1 and H3N2. DCAF7 acts as a substrate recognition receptor to form a complete E3 ligase with CRL4B to specifically degrade the PA subunit, reducing polymerase activity and limiting viral RNA synthesis. Activation of CUL4B by etoposide promoted PA degradation and inhibited IAV virulence *in vivo*. Our findings establish a working model to illustrate the function of DCAF7 during IAV infection (Fig. S10). In summary, our findings reveal a novel host anti-IAV mechanism, clarify that polymerase PA is regulated by host CRL4B^DCAF7^ E3 ligases during IAV infection, and demonstrate the possibility of etoposide for influenza treatment.

DCAF7 is a multifunctional protein that plays a role in several species, and it acts as a scaffold protein to control the cellular signaling by recruiting downstream proteins. Several reports have shown that DCAF7 interacts with several protein kinases and negatively regulates their signaling activity, including DYRK1A/B, HIPK2, and MEKK1 (52, 53). Another known function of DCAF7 is as a substrate recognition receptor that determines substrate specificity and forms intact E3 ligases with the CUL4-DDB1-RBX1 complex for target protein degradation (20). The function of DCAF7 in virus infection has been poorly studied. We found that DCAF7 acts as a substrate recognition receptor and CRL4B complex to form CRL4B^DCAF7^E3 ligase for ubiquitinating degradation of the IAV polymerase PA subunit. This is the first time that DCAF7 has been identified to regulate viral replication through its classical ubiquitination-degradation function in IAV infection, and the contribution of DCAF7 in host resistance to IAV infection was affirmed. Indeed, in addition to DCAF7, more than 60 DCAF (DDB1 and CUL4-associated factor) proteins have been identified (40). Whether they also play a role in influenza virus infection is unclear, but mass spectrometry data showed that they were not among the host factors that interact with influenza virus polymerase subunits with high confidence (31). This indicated that DCAF7 was relatively specific against influenza A virus replication.

PA protein is essential for viral transcription, and several studies have identified some host proteins that regulate PA. For instance, HDAC6 mediates the deacetylation of PA protein at the K664 site and inhibits the replication and transcription of IAV genome (38). HAX1 is a novel PA-binding protein and inhibits its nuclear accumulation by interacting with the PA nuclear localization signal (NLS) domain (36). During influenza A virus infection, the three polymerase subunits are ubiquitinated by host cells (37). Several E3 ligases that catalyze the ubiquitination of PB1 and PB2 subunits have been identified, but the E3 ligases that induce the ubiquitination of PA protein have not been reported (18, 54). In this work, we first identified CRL4B^DCAF7^ as an E3 ligase of PA and degraded it by inducing K48-linked polyubiquitination of PA at the K609 site. The K609 residue of PA is highly conserved among human, avian, and swine influenza strains (37). Moreover, DCAF7 is also highly conserved in human, chicken, and pig species, with 100% amino acid sequence similarity. This finding suggests that DCAF7 is an intrinsic broad-spectrum antiviral factor during human-, avian-, and swine-origin IAV infection. In summary, this work reveals the function and mechanism of the DCAF7-CRL4B axis in inhibiting influenza A virus infection and provides a theoretical basis for future anti-IAV drug design. It also demonstrated the anti-influenza activity of etoposide, providing a new treatment option for future influenza outbreaks.

## MATERIALS AND METHODS

### Cells and viruses

Human embryonic kidney 293T cells (HEK293T), human lung adenocarcinoma cells (A549), Madin-Darby canine kidney cells (MDCK), and human cervical cancer cells (HeLa) were cultured in Dulbecco’s Modified Eagle’s medium (DMEM, Biological Industries) containing 10% fetal bovine serum (FBS, Biological Industries) and 1% penicillin/streptomycin (Gibco) at 37 °C in a 5% CO_2_ incubator. The influenza A/Puerto Rico/8/1934 (PR8, H1N1), influenza PR8-*Renilla* luciferase reporter virus (PR8-Rluc), mouse-adapted influenza PR8 virus, and H3N2 virus were maintained in our laboratory. Viruses were propagated in MDCK cells or 10-day-old embryonated chicken eggs and stored at −80 °C until use.

### Antibodies and reagents

The following primary antibodies were used in this study: Mouse monoclonal anti-GFP (ABclonal, AE012), Rabbit monoclonal anti-Myc (ABclonal, AE070), Rabbit monoclonal anti-HA (Sigma, H6908), Rabbit monoclonal anti-Flag (Sigma, F7425), Mouse monoclonal anti-GAPDH (ABclonal, AC002), Mouse monoclonal anti-α-Tubulin (Sigma, T6199), Mouse monoclonal anti-GST (ABclonal, AE001), Mouse monoclonal anti-His (ABclonal, AE003), Rabbit polyclonal anti-PB1 (GeneTex, GTX125923), Rabbit polyclonal anti-PB2 (GeneTex, GTX125926), Rabbit polyclonal anti-PA (GeneTex, GTX125932), Rabbit monoclonal anti-DCAF7 (ABclonal, A9361), Rabbit polyclonal anti-IFITM3 (ABclonal, A13070), Mouse monoclonal anti-Hemagglutinin (Sino Biological, 11684-MM03), and Mouse Monoclonal anti-NP (Abcam, ab128193). The secondary antibodies used for Western blotting were goat anti-mouse and anti-rabbit IgG (Jackson, 115-035-174 and 111-005-144); the secondary antibodies used for immunofluorescence analysis were Alexa Fluor 555 goat anti-mouse IgG (Invitrogen, A-21422), Alexa Fluor 488 goat anti-mouse IgG (Invitrogen, A-11001), and Alexa Fluor 488 goat anti-rabbit IgG (Invitrogen, A-11094). The main reagents used in this study are as follows: MG132 (MCE, HY-13259), Chloroquine (MCE, HY-17589A), 3-MA (MCE, HY-19312), MLN4924 (APExBio, B1036), recombinant human IFN-α (Novoprotein, C005), recombinant human IFN-β (Peprotech, 300–02B.C.BC), and dual luciferase reporter assay kit (Promega, E1980).

### Plasmid construction and transfection

The open reading frames (ORFs) of human KPNA3, ALDH3A2, ACAD9, TIMM23B, PKP2, HAUS6, CDIPT, TECR, DNAJB6, EIF3D, CCT6B, SLC25A1, TTC27, DCAF7, MYCBP, BAG5, HAUS3, COPR5, PCBP1, PASK, BBX, ZNF281, HAUS8, SSR1, DNAJC16, ZCCHC8, NOP58, FAM120A, ADCK3, PAK4, UBE3C, SLC25A22, SPTLC1, and GNB1 were cloned into pCDH-CMV-Flag empty vector using specific primers. The cDNA clones of PR8 (H1N1) PB1, PB2, PA, NA, M1, M2, NS1, and NP were separately inserted into the pCMV-Flag empty vector. The Flag-HA (Hemagglutinin) plasmid was previously described (55). Flag-PB1 (1-265), Flag-PB1 (266-493), Flag-PB1 (494-757), Flag-PB2 (1-281), Flag-PB2 (282-522), Flag-PB2 (523-759), Flag-PA (1-259), Flag-PA (1-521), and Flag-PA (252-716) were generated using the pCMV-Flag-PB1/PB2/PA plasmids as amplification templates. All mutant plasmids of PA were derived from pCMV-Flag-PA using specific primers. The ORFs of human DCAF7 and DDB1 were cloned into the pCMV-Myc empty vector. The ORF of human RBX1 was cloned into the Bio-TEV-GFP empty vector. GST-DCAF7 and His-PB1/PB2/PA were constructed by inserting coding regions into the prokaryotic expression vectors pGEX-4T or PET-30A, respectively. All PCR primers in this study are listed in the supplemental table. All constructs were verified by sequencing. HEK293T, A549, and MDCK cells were transfected with plasmids using Neofect DNA transfection reagent (Neofect, TF20121201) or Lipofectamine 2000 (Thermo Fisher Scientific, 11668019).

### Establishment of stable cell lines overexpressing DCAF7

To obtain A549 and HEK293T cell lines that stably express DCAF7, HEK293T cells were co-transfected with the constructed lentiviral pCDH-CMV-Flag-IRES-Blast-DCAF7 plasmid or empty vector, Δ8.9 packaging plasmid, and pVSV-G plasmid. At 48 h post-transfection, viral supernatants were collected and used to transduce HEK293T or A549 cells. Finally, cells were screened with 25 µg/mL blasticidin (Sigma), and the expression of DCAF7 in both cells was detected by immunoblotting analysis with the DCAF7-specific antibody.

### Generation of DCAF7 knockout cell line by CRISPR/Cas9

DCAF7-knockout (KO) A549 cell line was established using the CRISPR/Cas9 system as described previously (56). The DCAF7 sgRNA sequences were synthesized and inserted into the lentiCRISPRv2-puro vector. Constructed lentiCRISPRv2 plasmids were transfected into HEK293T cells with the packaging plasmids to produce the recombined lentivirus. Monolayer A549 cells were transduced with lentivirus. At 48 h post-infection, the cells were selected with puromycin (2 µg/mL), and a single clonal cell line was obtained via limited dilution. The KO effect of DCAF7 was confirmed by immunoblotting analysis. All sgRNA sequences in this study were listed in the supplemental table.

### Co-immunoprecipitation and pull-down assays

Briefly, cells were collected and lysed with RIPA lysis buffer (Beyotime, P0013) containing protease inhibitor cocktail and PMSF. The lysates were harvested by centrifugation at 12,000 *g*, 4 °C for 15 min, and then incubated with M2 beads (Sigma) at 4 °C overnight. The beads were washed three times with RIPA lysis buffer, the precipitated proteins were separated and subjected to Western blotting. GST, GST-DCAF7, and His-PB1/PB2/PA protein were expressed in *E. coli* BL21 and purified for pull-down assay. In the GST pull-down assay, GST or GST-DCAF7 were incubated with glutathione-sepharose 4B for 4 h at 4 °C, and then the washed samples were incubated with purified His-PB1 or His-PA at 4 °C overnight. Followed by washing the beads three times, pull-down eluates were analyzed by Western blotting. In the His pull-down assay, His or His-PB2 was incubated with Ni-NTA resin beads for 4 h at 4 °C, and then the washed samples were incubated with purified GST-DCAF7 at 4 °C overnight. Followed by washing the beads three times, and pull-down eluates were analyzed by Western blotting.

### RNA isolation and quantitative real-time PCR

Total RNA was extracted using TRIzol reagent (Ambion, USA) and reverse transcribed to cDNA with HiScript III 1st Strand cDNA Synthesis Kit (Vazyme, China). Quantitative real-time PCR was performed using ChamQ SYBR qPCR Master Mix (Vazyme, China) according to the manufacturer’s instructions. As described previously, the abundances of influenza A virus NP vRNA, cRNA, and mRNA were detected by strand-specific RT-PCR (57). The mRNA level of the detected gene was normalized to GAPDH; specific primers for reverse transcription or qRT-PCR are listed in the supplemental table.

### Immunofluorescence assay

HeLa cells were transfected with the indicated plasmids or A549 cells were infected with the H1N1/H3N2 virus. Cells were washed with PBS and fixed with 4% paraformaldehyde for 15 min. Followed by cells that were permeabilized with 0.1% Triton X-100 (Sigma) for 15 min and blocked with 5% bovine serum albumin for 1 h. Cells were incubated with indicated primary antibodies at 4 °C overnight and stained with corresponding fluorescent-conjugated secondary antibodies at room temperature for 1 h. Finally, the nuclei were stained with DAPI (Beyotime, C1002) for 10 min and images were observed by fluorescence microscopy.

### Plaque assay

Virus titers were measured via plaque assay on MDCK cells as described previously (58). Monolayer MDCK cells were seeded on a 24-well plate and then infected with a series of 10-fold dilutions of viral supernatants for 1 h at 37 °C. After the viruses were removed, the cells were covered with agarose medium (2 × MEM containing 2 µg/mL of TPCK-trypsin, 0.2% BSA, and 1% low melting point agarose) and left at 4 °C until it is solid. Followed by the cells were cultured for 72 h at 37 °C. The agarose layer was removed, and the plaques were counted after staining with 1% crystal violet solution.

### IAV minigenome system for polymerase activity

Cells were transfected with pCAGGS-PB1 (100 ng), pCAGGS-PB2 (100 ng), pCAGGS-PA (100 ng), pCAGGS-NP (300 ng), pPolI-Luc (100 ng), pTK-RL (10 ng), and DCAF7 expression plasmid or empty vector or DCAF7 siRNAs. At the indicated transfection time point, cells were lysed, and the luciferase activity was measured with a dual luciferase reporter assay kit (Promega).

### Ubiquitination assay

For exogenous ubiquitination experiments, HEK293T cells were transfected with the indicated plasmids for 48 h. The cells were lysed, and cell lysates were immunoprecipitated with anti-Flag antibody. The precipitates were analyzed by Western blotting.

### Cell viability assay

Cell viability was detected by a Cell Counting Kit-8 (CCK-8) reagent (Abbkine, BMU106). To be specific, cells were transfected with the corresponding plasmids. Then, CCK-8 reagent was added to each well of a plate, and the cells were incubated at 37 °C for 1 h. The absorbance at 450 nm was measured by a microplate reader.

### Mouse infection experiments

Eight-week-old female C57BL/6J mice were administered orally with MLN4924 or etoposide (20 mg/kg). Mice were anesthetized with isoflurane and inoculated intranasally with 50 µL of PBS containing the mouse-adapted PR8 strain. The mock group was inoculated intranasally with an equal volume of PBS as a negative control. All the mice were monitored daily for weight changes and mortality. On the sixth day after infection, the mice were euthanized to obtain lung tissue samples for relevant detection.

### Statistical analysis

The results are representative of three independent assays, and data are presented as mean ± standard deviation (SD). The statistical significance was analyzed using the unpaired t-test of GraphPad Prism 8. *P* value > 0.05 means no significance, and *P* value < 0.05 means significance (*P < 0.05, **P < 0.01, ***P < 0.001, ****P < 0.0001).

## Data Availability

All the data from this study are available in the main text or supplemental material.

## SUPPLEMENTAL MATERIAL

Supplemental material jvi.00133-25-s0001.pdf Figures S1 to S10 and Table S1.

ASM does not own the copyrights to Supplemental Material that may be linked to, or accessed through, an article. The authors have granted ASM a non-exclusive, world-wide license to publish the Supplemental Material files. Please contact the corresponding author directly for reuse.

## References

[1] Salomon R, Webster RG. 2009. The influenza virus enigma. Cell 136:402–410. doi:10.1016/j.cell.2009.01.029 19203576 PMC2971533

[2] Allen JD, Ross TM. 2018. H3N2 influenza viruses in humans: viral mechanisms, evolution, and evaluation. Hum Vaccin Immunother 14:1840–1847. doi:10.1080/21645515.2018.1462639 29641358 PMC6149781

[3] Park J-E, Ryu Y. 2018. Transmissibility and severity of influenza virus by subtype. Infect Genet Evol 65:288–292. doi:10.1016/j.meegid.2018.08.007 30103034

[4] Hussain M, Galvin HD, Haw TY, Nutsford AN, Husain M. 2017. Drug resistance in influenza A virus: the epidemiology and management. Infect Drug Resist 10:121–134. doi:10.2147/IDR.S105473 28458567 PMC5404498

[5] Bosaeed M, Kumar D. 2018. Seasonal influenza vaccine in immunocompromised persons. Hum Vaccin Immunother 14:1311–1322. doi:10.1080/21645515.2018.1445446 29485353 PMC6037456

[6] Alexander ME, Bowman CS, Feng Z, Gardam M, Moghadas SM, Röst G, Wu J, Yan P. 2007. Emergence of drug resistance: implications for antiviral control of pandemic influenza. Proc Biol Sci 274:1675–1684. doi:10.1098/rspb.2007.0422 17507331 PMC2493585

[7] Handel A, Longini IM Jr, Antia R. 2009. Antiviral resistance and the control of pandemic influenza: the roles of stochasticity, evolution and model details. J Theor Biol 256:117–125. doi:10.1016/j.jtbi.2008.09.021 18952105 PMC2624577

[8] Bouvier NM, Palese P. 2008. The biology of influenza viruses. Vaccine (Auckl) 26:D49–D53. doi:10.1016/j.vaccine.2008.07.039 PMC3074182 19230160

[9] Eisfeld AJ, Neumann G, Kawaoka Y. 2015. At the centre: influenza A virus ribonucleoproteins. Nat Rev Microbiol 13:28–41. doi:10.1038/nrmicro3367 25417656 PMC5619696

[10] Noda T, Kawaoka Y. 2010. Structure of influenza virus ribonucleoprotein complexes and their packaging into virions. Rev Med Virol 20:380–391. doi:10.1002/rmv.666 20853340 PMC6029254

[11] Dias A, Bouvier D, Crépin T, McCarthy AA, Hart DJ, Baudin F, Cusack S, Ruigrok RWH. 2009. The cap-snatching endonuclease of influenza virus polymerase resides in the PA subunit. Nature 458:914–918. doi:10.1038/nature07745 19194459

[12] Blaas D, Patzelt E, Kuechler E. 1982. Identification of the cap binding protein of influenza virus. Nucleic Acids Res 10:4803–4812. doi:10.1093/nar/10.15.4803 7133998 PMC321130

[13] Biswas SK, Nayak DP. 1994. Mutational analysis of the conserved motifs of influenza A virus polymerase basic protein 1. J Virol 68:1819–1826. doi:10.1128/JVI.68.3.1819-1826.1994 8107244 PMC236644

[14] Plotch SJ, Bouloy M, Ulmanen I, Krug RM. 1981. A unique cap(m^7^GpppXm)-dependent influenza virion endonuclease cleaves capped RNAs to generate the primers that initiate viral RNA transcription. Cell 23:847–858. doi:10.1016/0092-8674(81)90449-9 6261960

[15] Sun N, Li C, Li X-F, Deng Y-Q, Jiang T, Zhang N-N, Zu S, Zhang R-R, Li L, Chen X, Liu P, Gold S, Lu N, Du P, Wang J, Qin C-F, Cheng G. 2020. Type-IInterferon-inducible SERTAD3 inhibits influenza A virus replication by blocking the assembly of viral RNA polymerase complex. Cell Rep 33:108342. doi:10.1016/j.celrep.2020.108342 33147462

[16] Zhou Y, Li T, Zhang Y, Zhang N, Guo Y, Gao X, Peng W, Shu S, Zhao C, Cui D, Sun H, Sun Y, Liu J, Tang J, Zhang R, Pu J. 2024. BAG6 inhibits influenza A virus replication by inducing viral polymerase subunit PB2 degradation and perturbing RdRp complex assembly. PLoS Pathog 20:e1012110. doi:10.1371/journal.ppat.1012110 38498560 PMC10977894

[17] Yang M-L, Chen Y-C, Wang C-T, Chong H-E, Chung N-H, Leu C-H, Liu F-T, Lai MMC, Ling P, Wu C-L, Shiau A-L. 2023. Upregulation of galectin-3 in influenza A virus infection promotes viral RNA synthesis through its association with viral PA protein. J Biomed Sci 30:14. doi:10.1186/s12929-023-00901-x 36823664 PMC9948428

[18] Fu B, Wang L, Ding H, Schwamborn JC, Li S, Dorf ME. 2015. TRIM32 senses and restricts influenza A virus by ubiquitination of PB1 polymerase. PLoS Pathog 11:e1004960. doi:10.1371/journal.ppat.1004960 26057645 PMC4461266

[19] de Vetten N, Quattrocchio F, Mol J, Koes R. 1997. The an11 locus controlling flower pigmentation in petunia encodes a novel WD-repeat protein conserved in yeast, plants, and animals. Genes Dev 11:1422–1434. doi:10.1101/gad.11.11.1422 9192870

[20] Jin J, Arias EE, Chen J, Harper JW, Walter JC. 2006. A family of diverse Cul4-Ddb1-interacting proteins includes Cdt2, which is required for S phase destruction of the replication factor Cdt1. Mol Cell 23:709–721. doi:10.1016/j.molcel.2006.08.010 16949367

[21] Yousefelahiyeh M, Xu J, Alvarado E, Yu Y, Salven D, Nissen RM. 2018. DCAF7/WDR68 is required for normal levels of DYRK1A and DYRK1B. PLoS One 13:e0207779. doi:10.1371/journal.pone.0207779 30496304 PMC6264848

[22] Peng Z, Liao Z, Matsumoto Y, Yang A, Tomkinson AE. 2016. Human DNA ligase I interacts with and is targeted for degradation by the DCAF7 specificity factor of the Cul4-DDB1 ubiquitin ligase complex. J Biol Chem 291:21893–21902. doi:10.1074/jbc.M116.746198 27573245 PMC5063974

[23] Frendo-Cumbo S, Li T, Ammendolia DA, Coyaud E, Laurent EMN, Liu Y, Bilan PJ, Polevoy G, Raught B, Brill JA, Klip A, Brumell JH. 2022. DCAF7 regulates cell proliferation through IRS1-FOXO1 signaling. iScience 25:105188. doi:10.1016/j.isci.2022.105188 36248734 PMC9556925

[24] Kawara H, Akahori R, Wakasugi M, Sancar A, Matsunaga T. 2019. DCAF7 is required for maintaining the cellular levels of ERCC1-XPF and nucleotide excision repair. Biochem Biophys Res Commun 519:204–210. doi:10.1016/j.bbrc.2019.08.147 31493872 PMC6759389

[25] Xu J, Ye Z, Zhuo Q, Gao H, Qin Y, Lou X, Zhang W, Wang F, Wang Y, Jing D, Fan G, Zhang Y, Chen X, Chen J, Xu X, Yu X, Ji S. 2023. MEN1 degradation induced by neddylation and the CUL4B-DCAF7 axis promotes pancreatic neuroendocrine tumor progression. Cancer Res 83:2226–2247. doi:10.1158/0008-5472.CAN-22-3599 36939378

[26] Li QJ, Fang XL, Li YQ, Lin JY, Huang CL, He SW, Huang SY, Li JY, Gong S, Liu N, Ma J, Zhao Y, Tang LL. 2024. DCAF7 acts as a scaffold to recruit USP10 for G3BP1 deubiquitylation and facilitates chemoresistance and metastasis in nasopharyngeal carcinoma. Adv Sci (Weinh) 11:e2403262. doi:10.1002/advs.202403262 38973296 PMC11423104

[27] Wang Q, Geng Z, Gong Y, Warren K, Zheng H, Imamura Y, Gao Z. 2018. WDR68 is essential for the transcriptional activation of the PRC1-AUTS2 complex and neuronal differentiation of mouse embryonic stem cells. Stem Cell Res 33:206–214. doi:10.1016/j.scr.2018.10.023 30448639

[28] Alvarado E, Yousefelahiyeh M, Alvarado G, Shang R, Whitman T, Martinez A, Yu Y, Pham A, Bhandari A, Wang B, Nissen RM. 2016. Wdr68 mediates dorsal and ventral patterning events for craniofacial development. PLoS One 11:e0166984. doi:10.1371/journal.pone.0166984 27880803 PMC5120840

[29] Kuppuswamy M, Subramanian T, Kostas-Polston E, Vijayalingam S, Zhao L, Varvares M, Chinnadurai G. 2013. Functional similarity between E6 proteins of cutaneous human papillomaviruses and the adenovirus E1A tumor-restraining module. J Virol 87:7781–7786. doi:10.1128/JVI.00037-13 23637414 PMC3700293

[30] Komorek J, Kuppuswamy M, Subramanian T, Vijayalingam S, Lomonosova E, Zhao L-J, Mymryk JS, Schmitt K, Chinnadurai G. 2010. Adenovirus type 5 E1A and E6 proteins of low-risk cutaneous beta-human papillomaviruses suppress cell transformation through interaction with FOXK1/K2 transcription factors. J Virol 84:2719–2731. doi:10.1128/JVI.02119-09 20053746 PMC2826030

[31] Wang L, Fu B, Li W, Patil G, Liu L, Dorf ME, Li S. 2017. Comparative influenza protein interactomes identify the role of plakophilin 2 in virus restriction. Nat Commun 8:13876. doi:10.1038/ncomms13876 28169297 PMC5309701

[32] Zheng H, Ma L, Gui R, Lin X, Ke X, Jian X, Ye C, Chen Q. 2022. G protein subunit β1 facilitates influenza A virus replication by promoting the nuclear import of PB2. J Virol 96:e0049422. doi:10.1128/jvi.00494-22 35604143 PMC9215228

[33] Su W-C, Hsu S-F, Lee Y-Y, Jeng K-S, Lai MMC. 2015. A nucleolar protein, ribosomal RNA processing 1 homolog B (RRP1B), enhances the recruitment of cellular mRNA in influenza virus transcription. J Virol 89:11245–11255. doi:10.1128/JVI.01487-15 26311876 PMC4645683

[34] Lin J-Y, Li M-L, Shih S-R. 2009. Far upstream element binding protein 2 interacts with enterovirus 71 internal ribosomal entry site and negatively regulates viral translation. Nucleic Acids Res 37:47–59. doi:10.1093/nar/gkn901 19010963 PMC2615614

[35] Fislová T, Thomas B, Graef KM, Fodor E. 2010. Association of the influenza virus RNA polymerase subunit PB2 with the host chaperonin CCT. J Virol 84:8691–8699. doi:10.1128/JVI.00813-10 20573828 PMC2919027

[36] Hsu W-B, Shih J-L, Shih J-R, Du J-L, Teng S-C, Huang L-M, Wang W-B. 2013. Cellular protein HAX1 interacts with the influenza A virus PA polymerase subunit and impedes its nuclear translocation. J Virol 87:110–123. doi:10.1128/JVI.00939-12 23055567 PMC3536397

[37] Günl F, Krischuns T, Schreiber JA, Henschel L, Wahrenburg M, Drexler HCA, Leidel SA, Cojocaru V, Seebohm G, Mellmann A, Schwemmle M, Ludwig S, Brunotte L. 2023. The ubiquitination landscape of the influenza A virus polymerase. Nat Commun 14:787. doi:10.1038/s41467-023-36389-0 36774438 PMC9922279

[38] Chen H, Qian Y, Chen X, Ruan Z, Ye Y, Chen H, Babiuk LA, Jung YS, Dai J. 2019. HDAC6 restricts influenza A virus by deacetylation of the RNA polymerase PA subunit. J Virol 93:e01896-18. doi:10.1128/JVI.01896-18 30518648 PMC6364008

[39] Chen Z, Sui J, Zhang F, Zhang C. 2015. Cullin family proteins and tumorigenesis: genetic association and molecular mechanisms. J Cancer 6:233–242. doi:10.7150/jca.11076 25663940 PMC4317758

[40] Lee J, Zhou P. 2007. DCAFs, the missing link of the CUL4-DDB1 ubiquitin ligase. Mol Cell 26:775–780. doi:10.1016/j.molcel.2007.06.001 17588513

[41] Pan Z-Q, Kentsis A, Dias DC, Yamoah K, Wu K. 2004. Nedd8 on cullin: building an expressway to protein destruction. Oncogene 23:1985–1997. doi:10.1038/sj.onc.1207414 15021886

[42] Li J-M, Jin J. 2012. CRL ubiquitin ligases and DNA damage response. Front Oncol 2:29. doi:10.3389/fonc.2012.00029 22655267 PMC3356132

[43] Brownell JE, Sintchak MD, Gavin JM, Liao H, Bruzzese FJ, Bump NJ, Soucy TA, Milhollen MA, Yang X, Burkhardt AL, et al.. 2010. Substrate-assisted inhibition of ubiquitin-like protein-activating enzymes: the NEDD8 E1 inhibitor MLN4924 forms a NEDD8-AMP mimetic in situ. Mol Cell 37:102–111. doi:10.1016/j.molcel.2009.12.024 20129059

[44] Baldwin EL, Osheroff N. 2005. Etoposide, topoisomerase II and cancer. Curr Med Chem Anticancer Agents 5:363–372. doi:10.2174/1568011054222364 16101488

[45] Yi J, Lu G, Li L, Wang X, Cao L, Lin M, Zhang S, Shao G. 2015. DNA damage-induced activation of CUL4B targets HUWE1 for proteasomal degradation. Nucleic Acids Res 43:4579–4590. doi:10.1093/nar/gkv325 25883150 PMC4482080

[46] Ma C, Li Y, Zong Y, Velkov T, Wang C, Yang X, Zhang M, Jiang Z, Sun H, Tong Q, Sun H, Pu J, Iqbal M, Liu J, Dai C, Sun Y. 2022. p21 restricts influenza A virus by perturbing the viral polymerase complex and upregulating type I interferon signaling. PLoS Pathog 18:e1010295. doi:10.1371/journal.ppat.1010295 35180274 PMC8920271

[47] Jahan AS, Biquand E, Muñoz-Moreno R, Le Quang A, Mok C-P, Wong HH, Teo QW, Valkenburg SA, Chin AWH, Man Poon LL, Te Velthuis A, García-Sastre A, Demeret C, Sanyal S. 2020. OTUB1 Is a key regulator of RIG-I-dependent immune signaling and is targeted for proteasomal degradation by influenza A NS1. Cell Rep 30:1570–1584. doi:10.1016/j.celrep.2020.01.015 32023470

[48] Yang H, Dong Y, Bian Y, Xu N, Wu Y, Yang F, Du Y, Qin T, Chen S, Peng D, Liu X. 2022. The influenza virus PB2 protein evades antiviral innate immunity by inhibiting JAK1/STAT signalling. Nat Commun 13:6288. doi:10.1038/s41467-022-33909-2 36271046 PMC9586965

[49] Javanian M, Barary M, Ghebrehewet S, Koppolu V, Vasigala V, Ebrahimpour S. 2021. A brief review of influenza virus infection. J Med Virol 93:4638–4646. doi:10.1002/jmv.26990 33792930

[50] Gaitonde DY, Moore FC, Morgan MK. 2019. Influenza: diagnosis and treatment. Am Fam Physician 100:751–758. 31845781

[51] McKimm-Breschkin JL. 2013. Influenza neuraminidase inhibitors: antiviral action and mechanisms of resistance. Influenza Other Respir Viruses 7:25–36. doi:10.1111/irv.12047 23279894 PMC4942987

[52] Ritterhoff S, Farah CM, Grabitzki J, Lochnit G, Skurat AV, Schmitz ML. 2010. The WD40-repeat protein Han11 functions as a scaffold protein to control HIPK2 and MEKK1 kinase functions. EMBO J 29:3750–3761. doi:10.1038/emboj.2010.251 20940704 PMC2989105

[53] Skurat AV, Dietrich AD. 2004. Phosphorylation of Ser640 in muscle glycogen synthase by DYRK family protein kinases. J Biol Chem 279:2490–2498. doi:10.1074/jbc.M301769200 14593110

[54] Sun N, Jiang L, Ye M, Wang Y, Wang G, Wan X, Zhao Y, Wen X, Liang L, Ma S, Liu L, Bu Z, Chen H, Li C. 2020. TRIM35 mediates protection against influenza infection by activating TRAF3 and degrading viral PB2. Protein Cell 11:894–914. doi:10.1007/s13238-020-00734-6 32562145 PMC7719147

[55] Yu L, Liu X, Wei X, Ren J, Wang X, Wu S, Lan K. 2024. C1QTNF5 is a novel attachment factor that facilitates the entry of influenza A virus. Virol Sin 39:277–289. doi:10.1016/j.virs.2024.01.003 38246238 PMC11074642

[56] Ran FA, Hsu PD, Wright J, Agarwala V, Scott DA, Zhang F. 2013. Genome engineering using the CRISPR-Cas9 system. Nat Protoc 8:2281–2308. doi:10.1038/nprot.2013.143 24157548 PMC3969860

[57] Kawakami E, Watanabe T, Fujii K, Goto H, Watanabe S, Noda T, Kawaoka Y. 2011. Strand-specific real-time RT-PCR for distinguishing influenza vRNA, cRNA, and mRNA. J Virol Methods 173:1–6. doi:10.1016/j.jviromet.2010.12.014 21185869 PMC3049850

[58] Zhang J, Huang F, Tan L, Bai C, Chen B, Liu J, Liang J, Liu C, Zhang S, Lu G, Chen Y, Zhang H. 2016. Host protein moloney leukemia virus 10 (MOV10) acts as a restriction factor of influenza A virus by inhibiting the nuclear import of the viral nucleoprotein. J Virol 90:3966–3980. doi:10.1128/JVI.03137-15 26842467 PMC4810528

