# Supplementary material for: Restriction of influenza A virus replication by host DCAF7-CRL4B axis
Source: J Virol. 2025 Mar 27;99(4):e00133-25. doi: 10.1128/jvi.00133-25 (PMC11998537; doi:10.1128/jvi.00133-25)
Supplement: Supplemental material — Figures S1 to S10 and Table S1. [file jvi.00133-25-s0001.pdf]

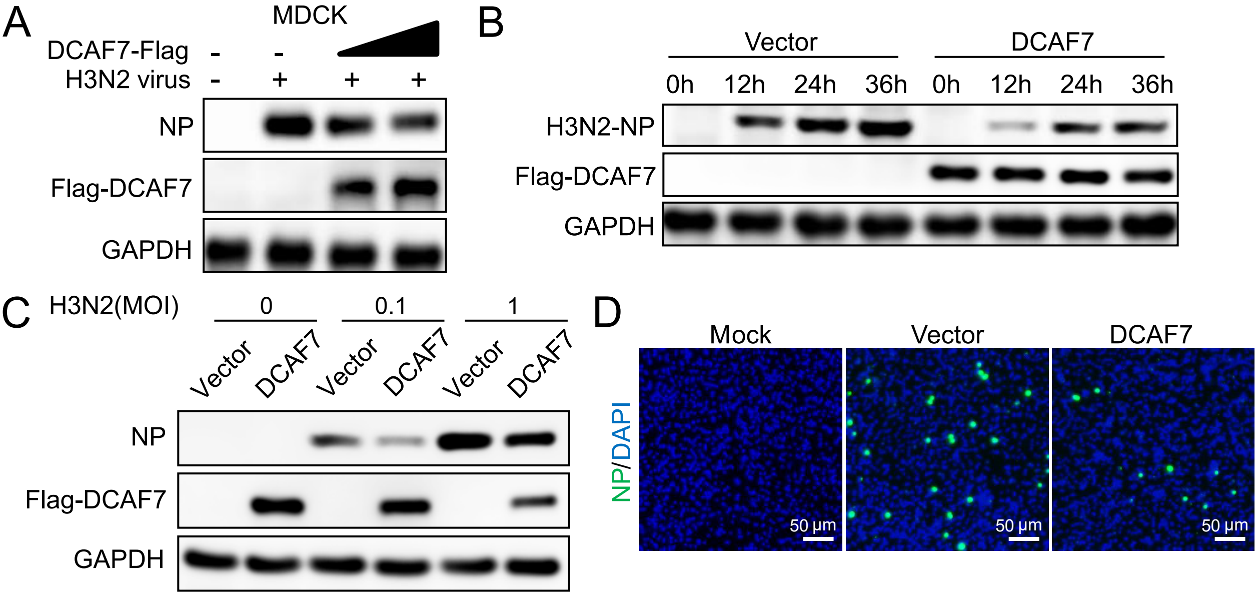

FIG S1 DCAF7 inhibits influenza A virus replication. (A) MDCK cells were transfected with flag-tagged DCAF7 protein expression plasmid (0, 0.5, 1 μg), and the viral NP protein level was examined after 24 h infection with H3N2 virus at an MOI of 0.02. (B) A549-DCAF7/A549-Vector cells were infected with H3N2 virus at an MOI of 0.2, the viral NP protein level was examined at the indicated time points. (C) 293T-DCAF7/293T-Vector cells were infected with H3N2 viruses (MOI=0, 0.1, 1) for 24 h. The viral NP protein level was examined by Western blotting. (D) A549-DCAF7/A549-Vector cells were infected with H3N2 virus at an MOI of 0.2 for 24 h. An immunofluorescence assay was performed, and then visualized by fluorescence microscopy (NP: green; Nuclear: blue), scale bar = 50 μm.

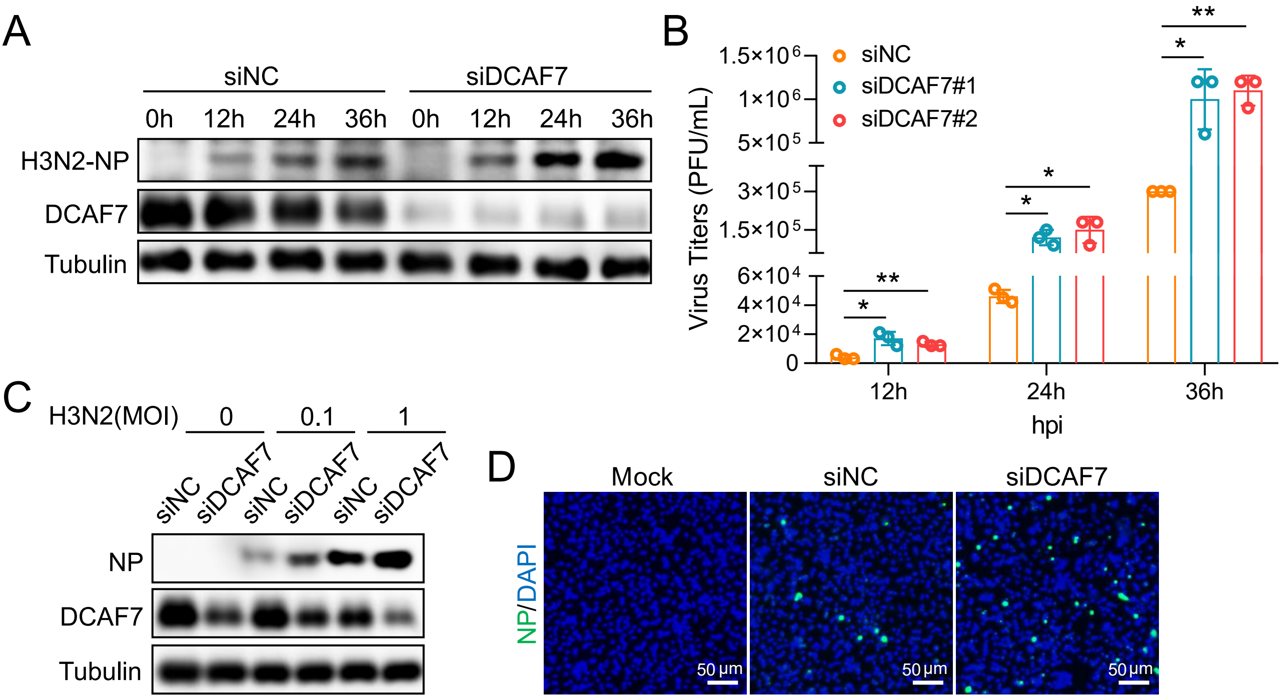

FIG S2 Knockdown of endogenous DCAF7 promotes influenza A virus replication. (A) A549 cells were transfected with siRNAs for 24 h, then infected with H3N2 viruses at an MOI of 0.1, the viral NP protein level was examined at the indicated time points. (B) A549 cells were transfected with siRNAs for 24 h, then infected with H3N2 viruses at an MOI of 0.1. The virus titers of cell supernatants were determined by plaque assays on MDCK cells. (C) A549 cells were transfected with siRNAs for 24 h and infected with H3N2 viruses (MOI=0, 0.1, 1) for 24 h. The viral NP protein level was examined by Western blotting. (D) A549 cells were transfected with siRNAs for 24 h, then infected with H3N2 viruses at an MOI of 0.1 for 24 h. An immunofluorescence assay was performed, and then visualized by fluorescence microscopy (NP: green; Nuclear: blue), scale bar = 50 μm. Unpaired t-test was used for data statistical analysis, and the data were shown as mean ± SD from three independent experiments. *P < 0.05, **P < 0.01.

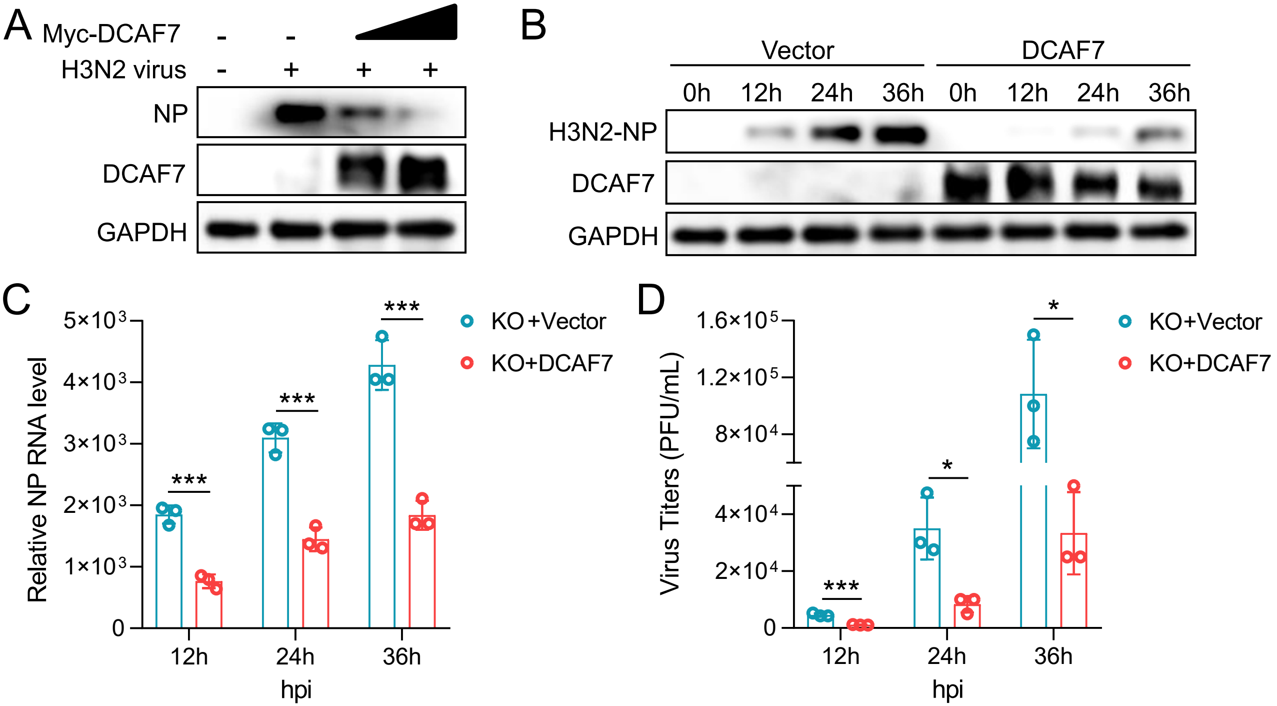

FIG S3 Restoration of DCAF7 expression inhibits influenza A virus replication. (A) The DCAF7-deficient A549 cell line was transfected with Myc-tagged DCAF7 protein expression plasmid (0, 0.5, 1 μg) for 24 h, and the viral NP protein level was examined after 24 h infection with H3N2 viruses at an MOI of 0.1. (B-D) The DCAF7-deficient A549 cell line was transfected with Myc-tagged DCAF7 protein expression plasmid or empty vector control (0.5 μg each) for 24 h, and the viral NP protein (B) and RNA (C) abundance were detected after infection with H3N2 (MOI=0.1). The virus titers of cell supernatants were determined by plaque assays on MDCK cells (D). Unpaired t-test was used for data statistical analysis, and the data were shown as mean ± SD from three independent experiments. $*P < 0.05$, $***P < 0.001$.

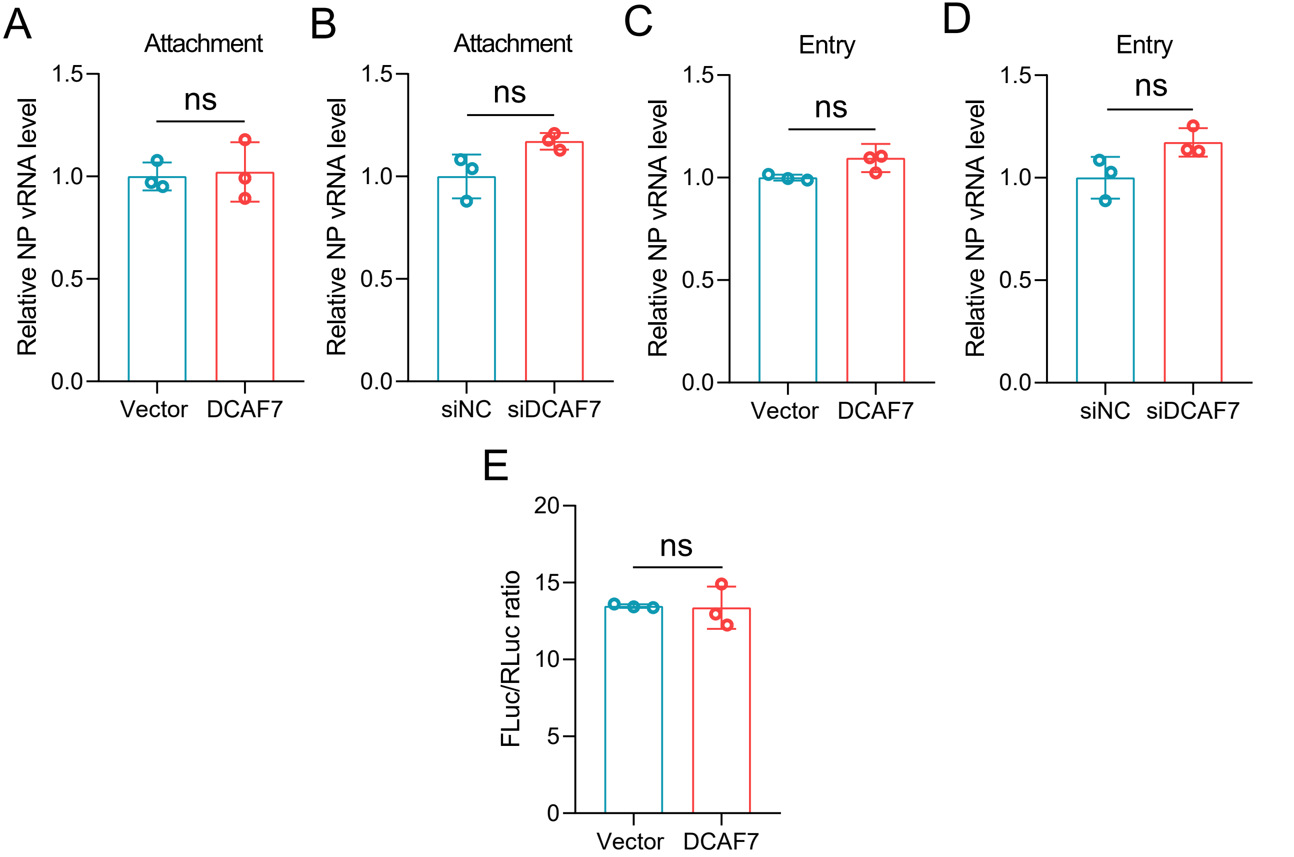

FIG S4 DCAF7 does not affect the attachment and entry of influenza A virus. (A and B) DCAF7 does not affect the attachment of influenza A virus. 293T-DCAF7/293T-Vector cells (A) or siRNA-treated A549 cells (B) were incubated with H1N1 (MOI=1) for 1 h at 4 ˚C, then cells were washed with PBS and lysed, the viral NP vRNA level was measured by qRT-PCR. (C and D) DCAF7 does not affect the entry of influenza A virus. 293T-DCAF7/293T-Vector cells (C) or siRNA-treated A549 cells (D) were incubated with H1N1 (MOI=1) for 1 h at 4 ˚C, then cells were washed with PBS and infected at 37 ˚C for another 30 min. The cells were washed twice with PBS and lysed, the viral NP vRNA level was measured by qRT-PCR. (E) DCAF7 does not affect the luciferase itself activity. 293T-DCAF7/293T-Vector cells were co-transfected with pRHF-EV-A71 5’ UTR (0.5 μg) and pTK-RL (10 ng) for 24 h, the luciferase activity was measured. Unpaired t-test was used for data statistical analysis, and the data were shown as mean ± SD from three independent experiments. ns, no significance.

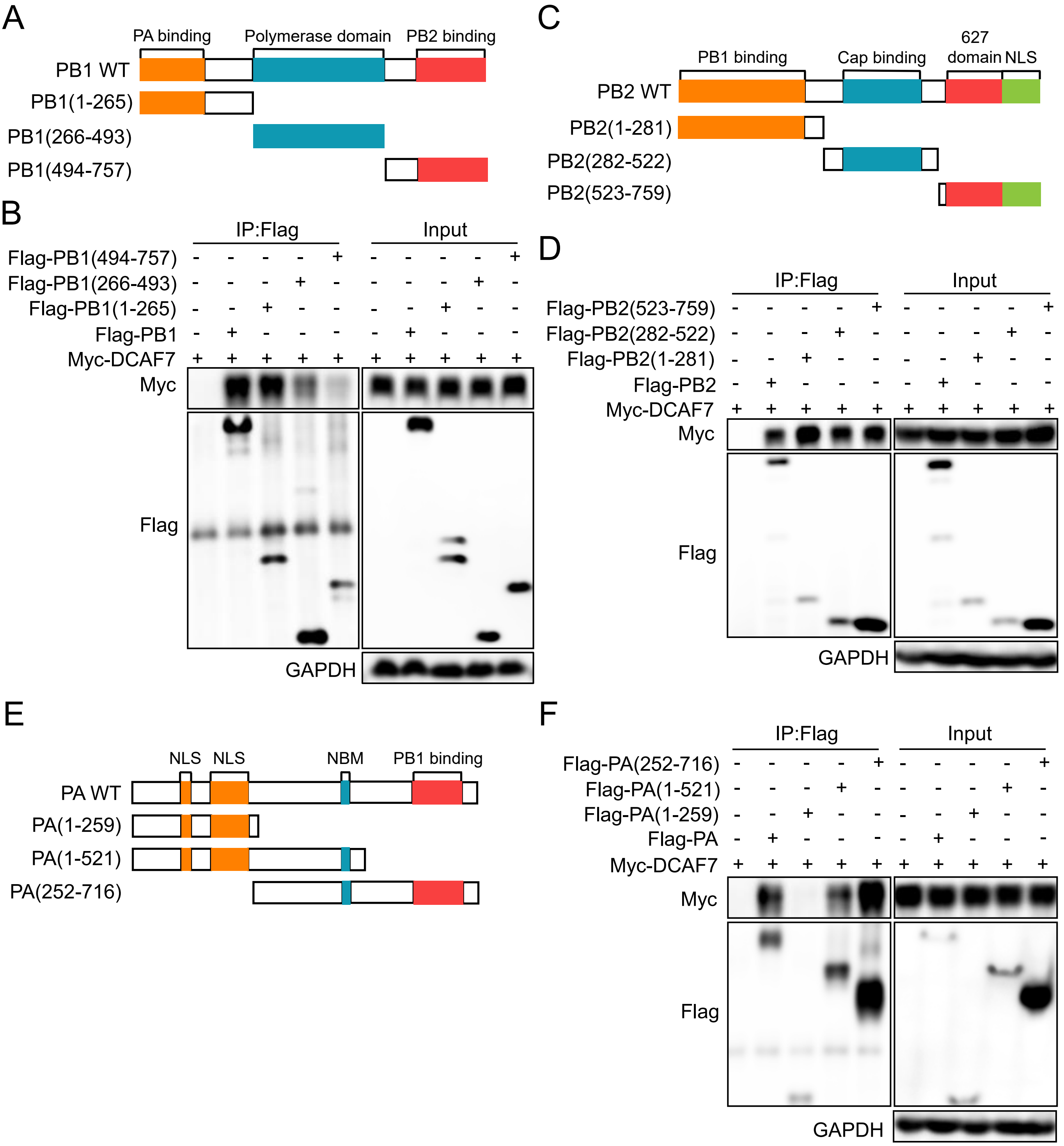

FIG S5 Mapping the domain of polymerase subunits responsible for interaction with DCAF7. (A) Schematic diagram of IAV PB1 domain and its truncation mutants. (B) Defining the DCAF7-interacting domain of PB1. HEK293T cells were transfected with Myc-tagged DCAF7 and Flag-tagged PB1 or its truncation mutants for 48 h, and then a co-immunoprecipitation (Co-IP) assay was performed with anti-Flag antibody. (C) Schematic diagram of IAV PB2 domain and its truncation mutants. (D) Defining the DCAF7-interacting domain of PB2. HEK293T cells were transfected with Myc-tagged DCAF7 and Flag-tagged PB2 or its truncation mutants for 48 h, and then a co-immunoprecipitation (Co-IP) assay was performed with anti-Flag antibody. (E) Schematic diagram of IAV PA domain and its truncation mutants. (F) Defining the DCAF7-interacting domain of PA. HEK293T cells were transfected with Myc-tagged DCAF7 and Flag-tagged PA or its truncation mutants for 48 h, and then a co-immunoprecipitation (Co-IP) assay was performed with anti-Flag antibody.

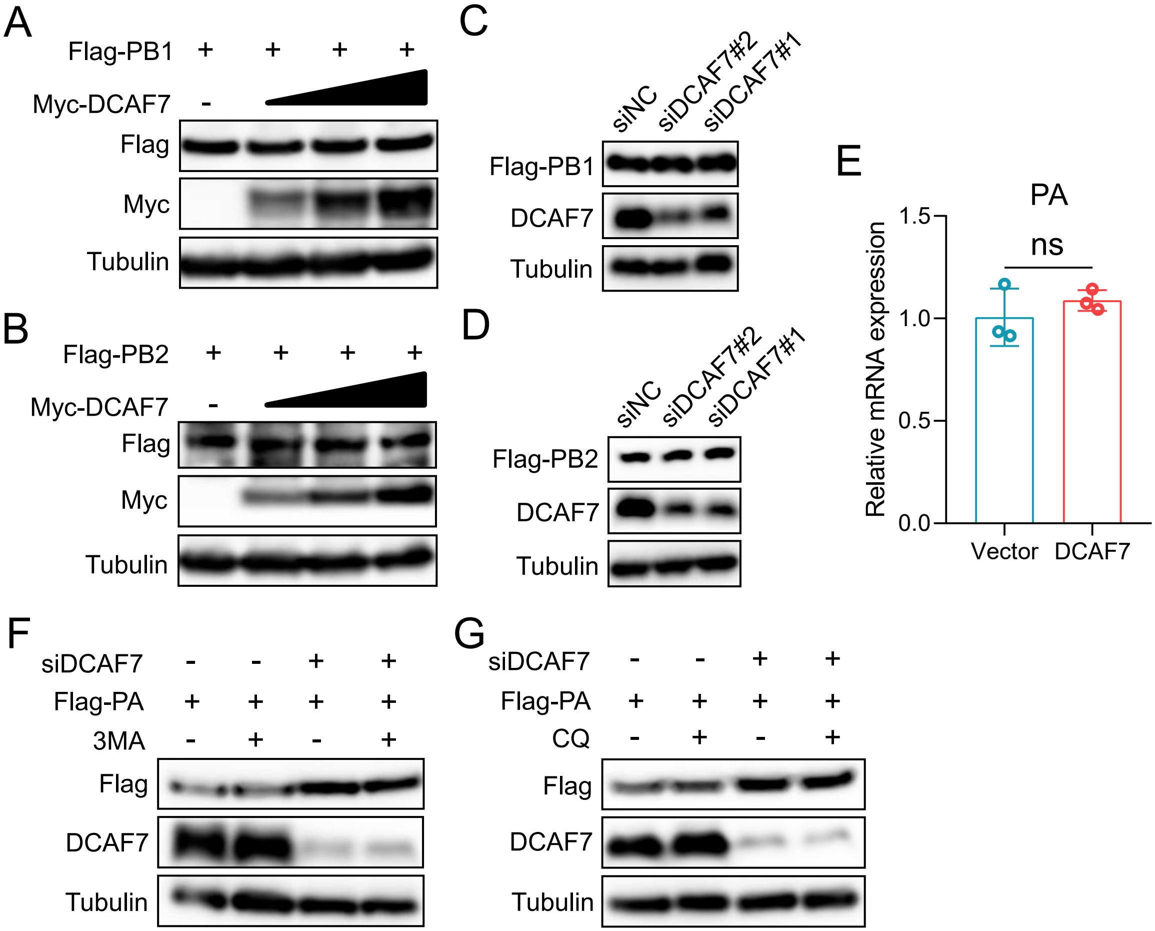

FIG S6 DCAF7 does not affect the protein level of PB1 and PB2. (A and B) HEK293T cells were co-transfected with Myc-tagged DCAF7 plasmid (0, 0.5, 1, 2 μg) and Flag-tagged PB1 (A) or Flag-tagged PB2 (B) plasmid (1 μg) for 48 h, the expression of viral polymerase subunits and DCAF7 were examined by Western blotting. (C and D) HEK293T cells were co-transfected with siRNAs targeting DCAF7 or negative control (NC), Flag-tagged PB1 (C) or Flag-tagged PB2 (D) plasmid (1 μg) for 36 h. The expression of viral polymerase subunits and DCAF7 were examined by Western blotting. (E) DCAF7 does not affect the mRNA expression of PA. HEK293T cells were transfected with the indicated plasmids for 36 h, and the mRNA expression of PA was measured by qRT-PCR. (F and G) HEK293T cells were transfected with siRNA targeting DCAF7 or negative control (NC) and Flag-tagged PA plasmid for 36 h, and then the cells were treated with or without 3MA (0.5 mM) (F) or CQ (50 μM) (G) for 12 h. The cells were lysed and cell lysates were detected. Unpaired t-test was used for data statistical analysis, and the data were shown as mean ± SD from three independent experiments, ns, no significance.

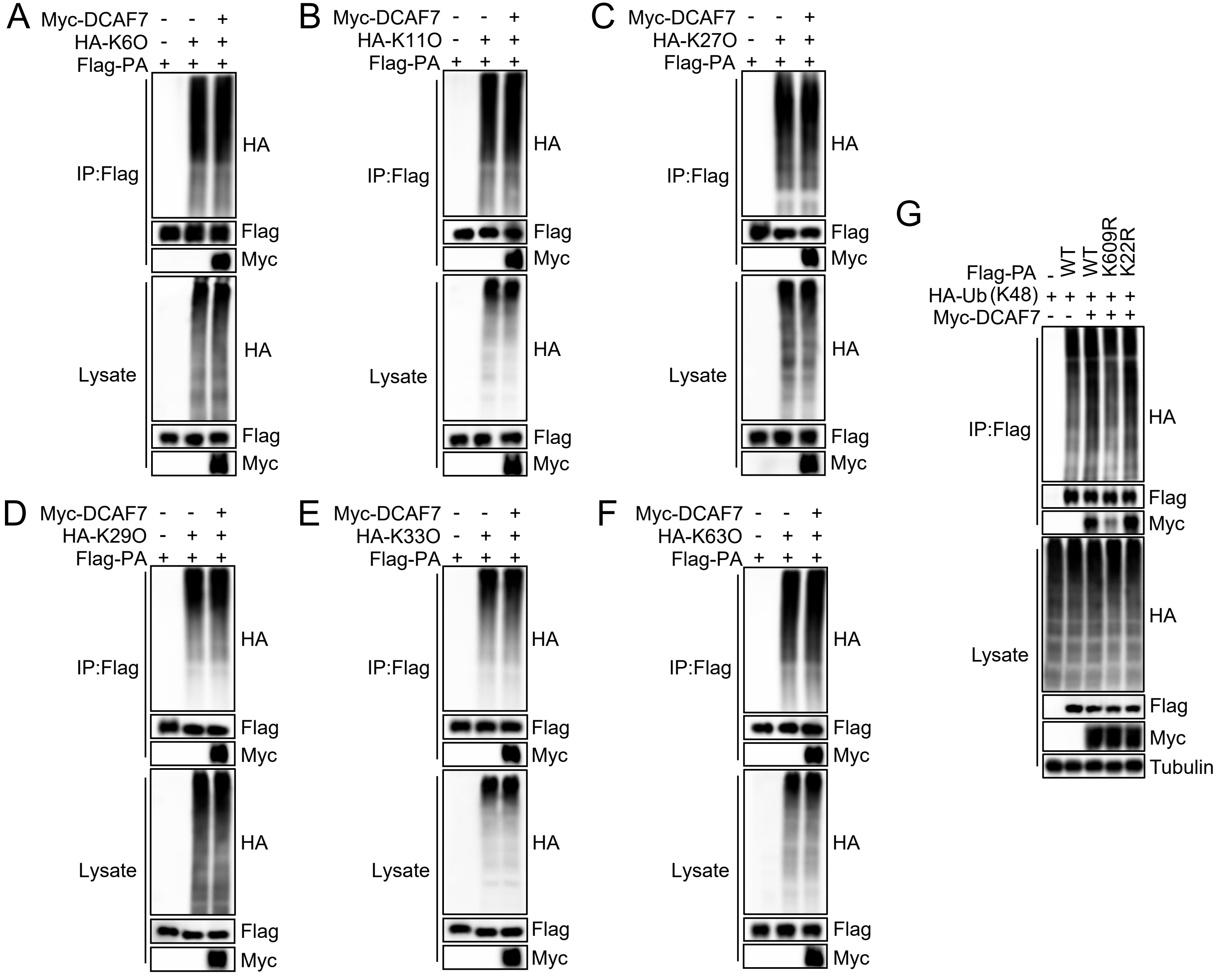

FIG S7 DCAF7 does not affect K6/K11/K27/K29/K33/K63-linked polyubiquitination of PA. (A-F) HEK293T cells were co-transfected with HA-K6O (A), HA-K11O (B), HA-K27O (C), HA-K29O (D), HA-K33O (E), HA-K63O (F), Myc-DCAF7, and Flag-PA plasmids for 48 h, and then immunoprecipitation assays were performed with anti-Flag antibody. KO, K only. (G) HEK293T cells were co-transfected with Myc-DCAF7, HA-Ub (K48), and PA-WT/K609R/K22R plasmids for 48 h, and then an immunoprecipitation assay was performed with anti-Flag antibody.

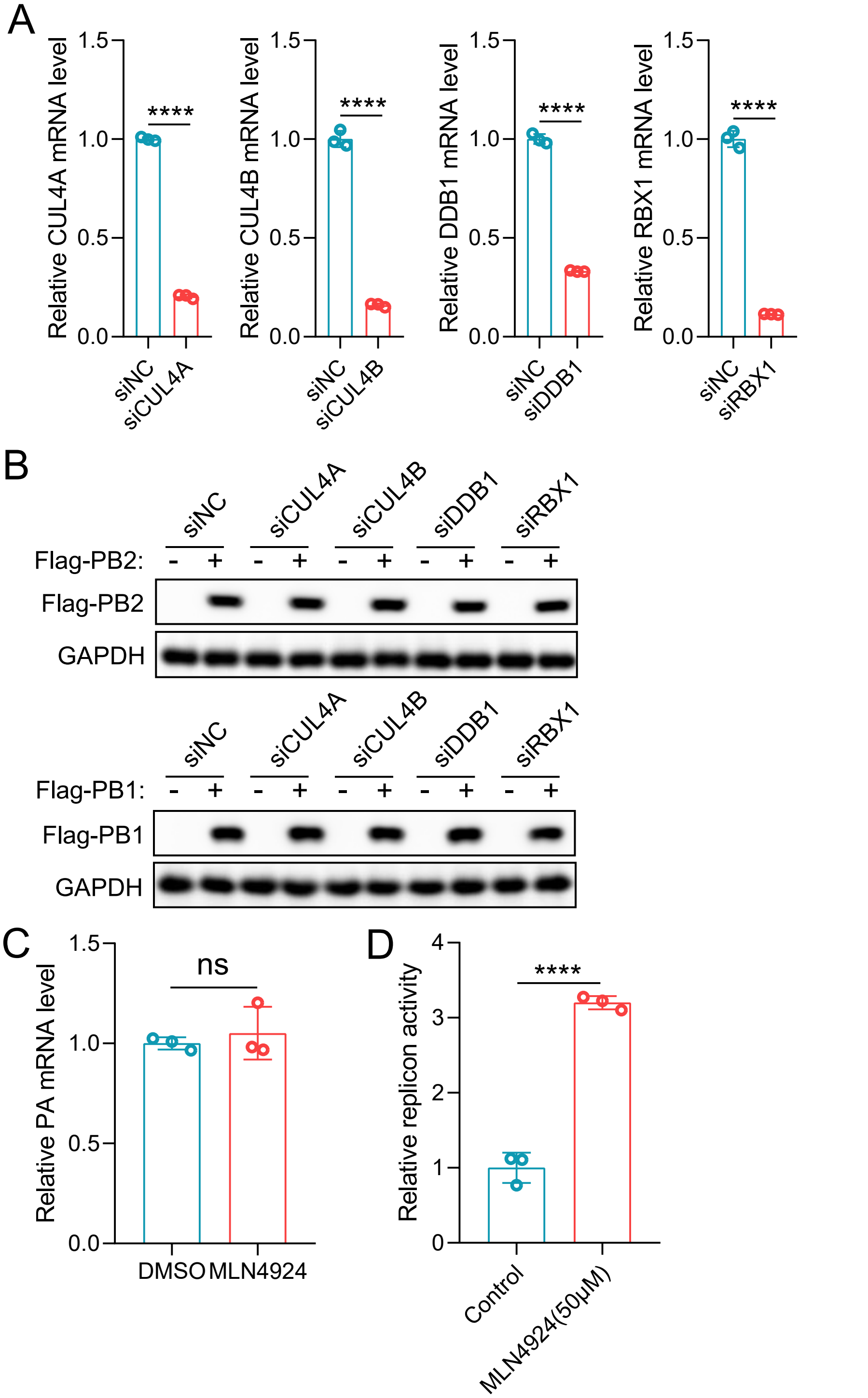

FIG S8 CUL4A, CUL4B, DDB1, and RBX1 does not affect the protein level of PB1 and PB2. (A) CUL4A, CUL4B, DDB1, and RBX1 were knocked down separately in HEK293T cells by siRNAs, and the knockdown efficiency of CUL4A, CUL4B, DDB1, and RBX1 were detected by qRT-PCR. (B) CUL4A, CUL4B, DDB1, and RBX1 were knocked down separately in HEK293T cells by siRNAs, and the knockdown cells were transfected with Flag-PB2, Flag-PB1, or empty vector for 48 h. The expression of PB2 and PB1 were analyzed by Western blotting. (C) HEK293T cells were transfected with Flag-PA for 36 h and treated with MLN4924 (50 μM) for 12 h, the mRNA level of PA was measured. (D) HEK293T cells were transfected with IAV minireplicon system plasmids for 36 h and treated with MLN4924 (50 μM) for 12 h, the luciferase activity was measured. Unpaired t-test was used for data statistical analysis, and the data were shown as mean ± SD from three independent experiments. ns, no significance, ****$P < 0.0001$.

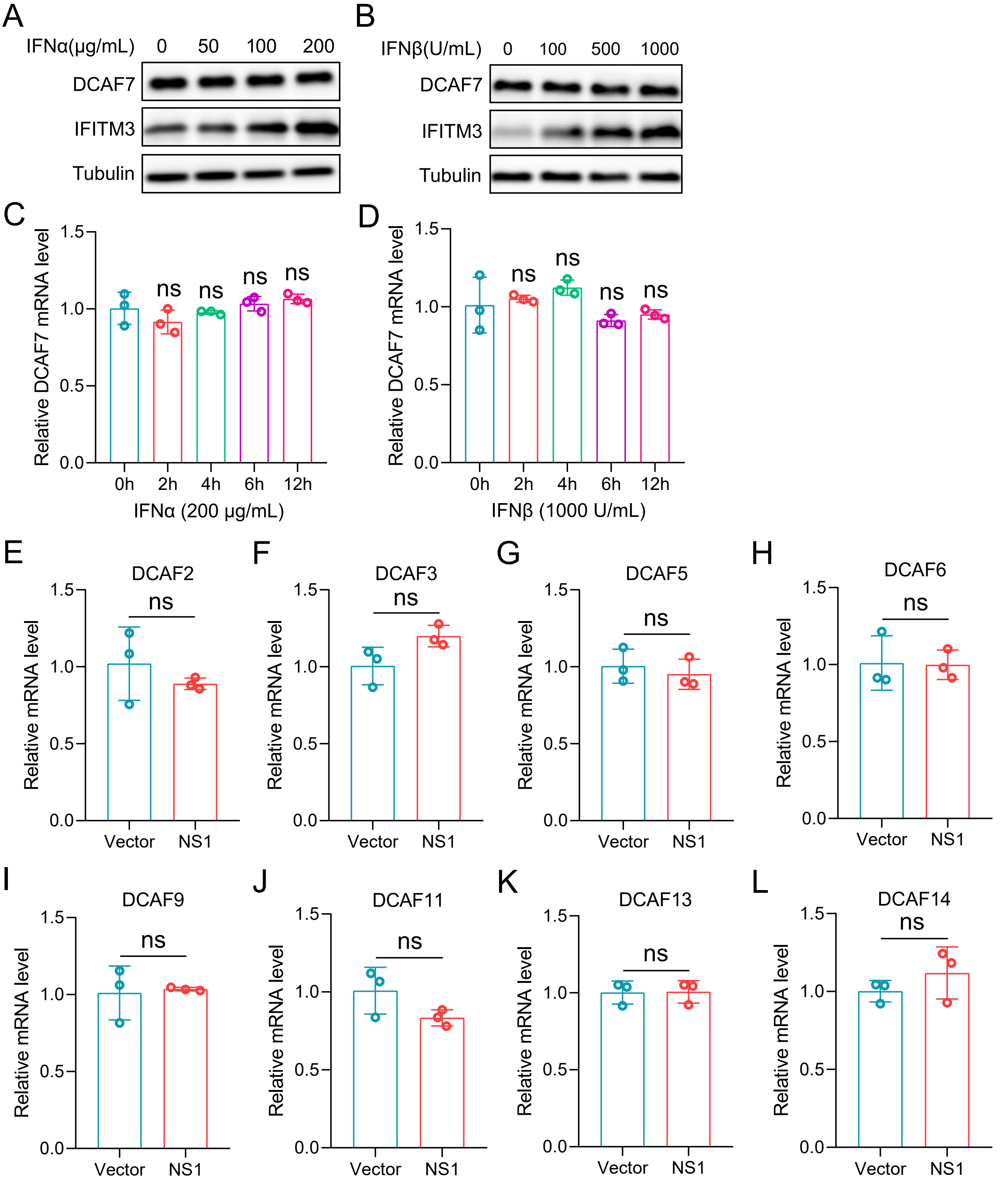

FIG S9 DCAF7 expression is not modulated by IFN and IAV NS1 does not affect the expression of other proteins in the DCAF family. (A and B) A549 cells were treated with the indicated concentration of IFNα (A) or IFNβ (B) for 6 h, and DCAF7 protein levels were detected by Western blotting with the indicated antibodies. (C and D) A549 cells were treated with IFNα (C) or IFNβ (D), and DCAF7 mRNA levels were detected by qRT-PCR. (E-L) IAV NS1 does not affect the expression of other proteins in the DCAF family. HEK293T cells were transfected with Flag-tagged viral NS1 protein expression plasmid or empty vector control (1.5 μg) for 48 h, and the mRNA level of DCAF2/DCAF3/DCAF5/DCAF6/DCAF9/DCAF11/DCAF13/DCAF14 was detected by qRT-PCR. Unpaired t-test was used for data statistical analysis, and the data were shown as mean ± SD from three independent experiments, ns, no significance.

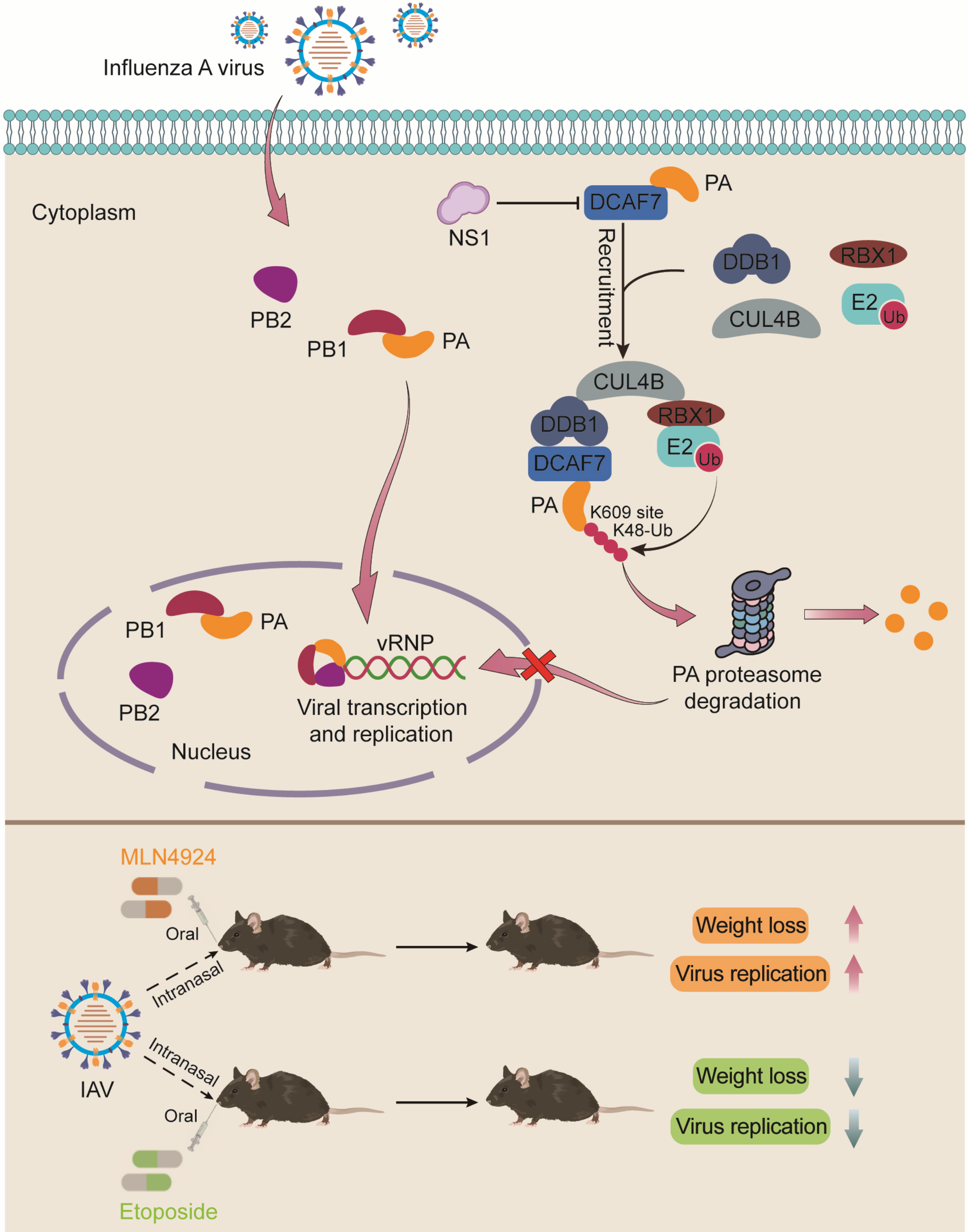

FIG S10 Working model for DCAF7 functions as an antiviral factor to inhibit IAV replication.

Supplementary table. All PCR and qPCR Primers, siRNAs, sgRNAs used in this study.

Gene	Purpose	Sequence (5'-3')
pCDH-Flag-KPNA3	Cloning	F:CCGCTCGAGATGGCCGAGAACCCCAGCTTG R:TCCCCCGGGATAAAATTAAATTCTTTTGTTTGAAGG
pCDH-Flag-ALDH3A2	Cloning	F:CCGCTCGAGATGGAGCTCGAAGTCCGGCG R:CGCGGATCCTCTCTGCTTACTGGACCAACGCAG
pCDH-Flag-ACAD9	Cloning	F:CCGGAATTCATGAGCGGCTGCGGGCTC R:TCCCCCGGGATGCATGTCCTGTCCAGAGGGTG
pCDH-Flag-TIMM23B	Cloning	F:CCGCTCGAGATGGAAGGAGGCGGGGGAAG R:TCCCCCGGGATGGAGCCAGACAGCAGCACACAGAAC
pCDH-Flag-PKP2	Cloning	F:ctaccggactcagatctcgagATGGCAGCCCCCGGCGCC R:ctgagggtggctccaggatccGTCTTTAAGGGAGTGGTAGGCT
pCDH-Flag-HAUS6	Cloning	F:CCGCTCGAGATGAGCTCGGCCTCGGTCAC R:CGCGGATCCTCTTGTCAAGTCAGACGGTGGTTC
pCDH-Flag-CDIPT	Cloning	F:CCGGAATTCATGCCAGACGAAAATATCTTCCTG R:TCCCCCGGGATCTTCTTCTTGGCGCGGTCTGC
pCDH-Flag-TECR	Cloning	F:CCGCTCGAGATGAAGCATTACGAGGTGGAG R:TCCCCCGGGATGAGCAGGAAGGGGATGATGGGC
pCDH-Flag-DNAJB6	Cloning	F:CCGCTCGAGATGGTGGATTACTATGAAG R:CGCGGATCCGTGATTGCCTTTGGTCGAC
pCDH-Flag-EIF3D	Cloning	F:CCGCTCGAGATGGCAAAGTTCATGACACCC R:CGCGGATCCAGTTTCTTCCTCTTCTTCTTCCTC
pCDH-Flag-CCT6B	Cloning	F:CCGCTCGAGATGGCTGCGATAAAGGCC R:CGCGGATCCTTTGAGAGAAGACATCCCAGCTC
pCDH-Flag-SLC25A1	Cloning	F:CCGCTCGAGATGTTCCCCGCGGCACTG R:CGCGGATCCGTCCGTCTTCCACACTTTGTTGAG
pCDH-Flag-TTC27	Cloning	F:CCGCTCGAGATGTGGACCCCGGAGCTG R:CGCGGATCCATACTGATTTCGAAACTGGTTGC
pCDH-Flag-DCAF7	Cloning	F:CCGGAATTCATGTCCCTGCACGGCAAACG R:TCCCCCGGGATCACTCTGAGTATCTCCAGGCAGTTG
pCDH-Flag-MYCBP	Cloning	F:CCGCTCGAGATGGCCCATTACAAAGCCGC R:TCCCCCGGGATTTCAGCACGCTTCTCCTCCTG
pCDH-Flag-BAG5	Cloning	F:CCGCTCGAGATGGATATGGGAAACCAACATC R:CGCGGATCCGTACTCCCATTCATCAGATTTCAGG
pCDH-Flag-HAUS3	Cloning	F:CCGCTCGAGATGAGTTGTGGAAATGAGTTTG R:CGCGGATCCATCTTCAAGACTAACAGCC
pCDH-Flag-COPR5	Cloning	F:CCGCTCGAGATGGACCTTCAGGCCGCC R:CGCGGATCCATCTTCAGCATCGTCAAACTGTC
pCDH-Flag-PCBP1	Cloning	F:CCGCTCGAGATGGATGCCGGTGTGACTG R:TCCCCCGGGATGCTGCACCCCATGCCCTTC
pCDH-Flag-PASK	Cloning	F:ctaccggactcagatctcgagATGGAGGACGGGGGCTTAAC R:ctgagggtggctccaggatccGCTGGTCAGCAGACGGGGATC
pCDH-Flag-BBX	Cloning	F:CCGCTCGAGATGAAAGGCAGTAATAGAAATAAGG R:TCCCCCGGGATCTGGTCAGCGCAGGAAATAAGTAC
pCDH-Flag-ZNF281	Cloning	F:CCGCTCGAGATGAAAATCGGCAGTGGGTTCC R:TCCCCCGGGATCCTGTAACTCTGGCTGGTGGGTG
pCDH-Flag-HAUS8	Cloning	F:CCGCTCGAGATGGCGGATTCCTCGGGG R:CGCGGATCCTGACAAGTCCCTCCCTGAACGAG
pCDH-Flag-SSR1	Cloning	F:CCGCTCGAGATGAGACTCCTCCCCCGC R:TCCCCCGGGATCTCATCAGATCCCACTGATCTC
pCDH-Flag-DNAJC16	Cloning	F:ctaccggactcagatctcgagATGGAAGTGAGAAAGTTGAGC R:ctgagggtggctccaggatccGTCTAGTTCAGGCCATGATG
pCDH-Flag-ZCCHC8	Cloning	F:CCGCTCGAGATGGCCGCAGAGGTGTATTTTG R:CGCGGATCCTTCAGAGGCCTTTTTGTTTTTC
pCDH-Flag-NOP58	Cloning	F:CCGCTCGAGATGTTGGTGCTGTTTGAAACGTCTG R:TCCCCCGGGATATCCTCGTTCTCTCTCTTTTTTTTC
pCDH-Flag-FAM120A	Cloning	F:ctaccggactcagatctcgagATGGGCGTGCAGGGCTTC R:ctgagggtggctccaggatccCTCTTCTTTATTTAAGACAGCTG
pCDH-Flag-ADCK3	Cloning	F:CCGCTCGAGATGGCTGCCATATTGGGAGACAC R:CGCGGATCCCTGCTGGGCCTGCCTCTTGC
pCDH-Flag-PAK4	Cloning	F:CCGCTCGAGATGTTTGGGAAGAGGAAGAAGC R:CGCGGATCCTCTGGTGCGGTTCTGGCG
pCDH-Flag-UBE3C	Cloning	F:ctaccggactcagatctcgagATGTTCAGCTTCGAAGGCGAC R:ctgagggtggctccaggatccGCTCAGCTCAAAGCCAGCG
pCDH-Flag-SLC25A22	Cloning	F:CCGCTCGAGATGGCTGATAAGCAGATCAGCC R:TCCCCCGGGATGGCCTGGGGGTCCTGCAGC
pCDH-Flag-SPTLC1	Cloning	F:CCGGAATTCATGGCGACCGCCACGGAG R:TCCCCCGGGATGAGCAGGACGGCCTGGGCTAC
pCDH-Flag-GNB1	Cloning	F:CCGCTCGAGATGAGTGAGCTTGACCAGTTACGGC R:CGCGGATCCGTTCCAGATCTTGAGGAAGCTATCC
pCMV-Myc-DCAF7	Cloning	F:GGGGTACCAACTTCTGACCTAATTGTTCCCGC R:CCGCTCGAGCACTCTGAGTATCTCCAGGCAGTTG
pCMV-Myc-DDB1	Cloning	F:ATTTGCGGCCGCATGTCGTACAACTACGTGGTAAC R:GCGTCGACATGGATCCGAGTTAGCTCCTC
GFP-RBX1	Cloning	F:GCGTCGACATGGCGGCAGCGATGGATG R:CGCGGATCCGTGCCCATACTTTTGGAATTCCC
pCMV-Flag-PB1	Cloning	F:CCAAGCTTATGGATGTCAATCCGACC R:GGGGTACCCTATTTTTGCCGTCTGAGC
pCMV-Flag-PB1 (1-265 aa)	Cloning	F:CCAAGCTTATGGATGTCAATCCGACTTTAC R:GGGGTACC CTATTTCTCACATATACTCCTTGC
pCMV-Flag-PB1 (266-493 aa)	Cloning	F:CCAAGCTT ATGCTTGAACAATCAGGATTGC R:GGGGTACCCTATGTGAATTCAAATGTACCTG
pCMV-Flag-PB1 (494-757 aa)	Cloning	F:CCAAGCTTATGAGTTTTTTCTACCGTTATGG R:GGGGTACCCTATTTTTGCCGTCTGAGCTC
pCMV-Flag-PB2	Cloning	F:GCGTCGACCATGGAAAGAATAAAAGAACTACG R:GGGGTACCATTGATGGCCATCCGAATTC
pCMV-Flag-PB2 (1-281 aa)	Cloning	F:GCGTCGACCATGGAAAGAATAAAAGAACTACGG R:GGGGTACCCTACAATAAAGATGCTAGTGGATC
pCMV-Flag-PB2 (282-522 aa)	Cloning	F:GCGTCGACCATGGAGATGTGCCACAGCAC R:GGGGTACCCTACTGTGTTTCACTGACCTCCTC
pCMV-Flag-PB2 (523-759 aa)	Cloning	F:GCGTCGACCATGGGAACAGAGAAACTGAC R:GGGGTACCCTAATTGATGGCCATCCG
pCMV-Flag-PA	Cloning	F:GCGTCGACCATGGAAGATTTTGTGCGAC R:GGGGTACCCTAACTCAATGCATGTGTAAGG
pCMV-Flag-PA (1-259 aa)	Cloning	F:GCGTCGACCATGGAAGATTTTGTGCGAC R:GGGGTACCAGGTTCAATTCTAGCATTTACTTC
pCMV-Flag-PA (1-521 aa)	Cloning	F:GCGTCGACCATGGAAGATTTTGTGCGAC R:GGGGTACCCACAAAGTTTACCACGTCGG
pCMV-Flag-PA (252-716 aa)	Cloning	F:GCGTCGACCATGGAAGTAAATGCTAGAATTGAAC R:GGGGTACCCTAACTCAATGCATGTGTAAGG
pCMV-Flag-NA	Cloning	F:GCGTCGACCATGAATCCAAATCAGAAA R:GGGGTACCCTTGTCAATGCTGAATGG
pCMV-Flag-M1	Cloning	F:GCGTCGACCATGAGTCTTCTAACCGAGGTC R:GGGGTACCCTTGAACCGTTGCATCTGC
pCMV-Flag-M2	Cloning	F:CGGAATTCATATGAGTCTTCTAACCGAGGTCG R:CCATCGATCTCCAGCTCTATGCTGACAAAATG
pCMV-Flag-NS1	Cloning	F:GCGTCGACCATGGATCCAAACACTGTGTCAAGC R:GGGGTACCAACTTCTGACCTAATTGTTCCCGC
pCMV-Flag-NP	Cloning	F:CGGAATTCATATGGCGTCTCAAGGCACC R:CCATCGATATTGTCGTACTCCTCTGCATTG
GST-DCAF7	Cloning	F:CCGGAATTCATGTCCCTGCACGGCAAACG R:CCGCTCGAGCACTCTGAGTATCTCCAGGCAG
His-PB1	Cloning	F:GGGGTACCATGGATGTCAATCCGACC R:CCGCTCGAGTTTTTGCCGTCTGAGC
His-PB2	Cloning	F:GGGGTACCATGGAAAGAATAAAAGAACTACG R:CCGCTCGAGATTGATGGCCATCCGAATTC
His-PA	Cloning	F:GGGGTACCATGGAAGATTTTGTGCGAC R:CCGCTCGAGACTCAATGCATGTGTAAGG
pCMV-Flag-PA-K22R	Mutation	F:CGGAAAAGGCAATGAGAGAGTATGGAGAGGA R:CTCATTGCCTTTTCCGCAAGCTCGAC
pCMV-Flag-PA-K29R	Mutation	F:ATGGAGAGGACCTGAGAATCGAAACAAACAA R:CTCAGGTCCTCTCCATACTCTTTCAT
pCMV-Flag-PA-K102R	Mutation	F:CTACAGGGGCTGAGAGACCAAAGTTTCTACC R:CTCTCAGCCCCTGTAGTGTTGCAAAT
pCMV-Flag-PA-K104R	Mutation	F:GGGCTGAGAAACCAAGGTTTCTACCAGATTT R:CTTGGTTTCTCAGCCCCTGTAGTGTT
pCMV-Flag-PA-K113R	Mutation	F:ATTTGTATGATTACAGGGAGAATAGATTCAT R:CTGTAATCATACAAATCTGGTAGAAA
pCMV-Flag-PA-K134R	Mutation	F:TATACTATCTGGAAAGGGCCAATAAAATTAA R:CTTTCCAGATAGTATATGTGAACTTC
pCMV-Flag-PA-K158R	Mutation	F:AGGAAATGGCCACAAGGGCCGACTACACTCT R:CTTGTGGCCATTTCCTCCCCAGTGAA
pCMV-Flag-PA-K213R	Mutation	F:CAGGAACAATGCGCAGGCTTGCCGACCAAAG R:CTGCGCATTGTTCCTGTGATTTCAAA
pCMV-Flag-PA-K245R	Mutation	F:GCTACATTGAGGGCAGGCTTTCTCAAATGTC R:CTGCCCTCAATGTAGCCGTTCGGTTC
pCMV-Flag-PA-K251R	Mutation	F:TTTCTCAAATGTCCAGAGAAGTAAATGCTAG R:CTGGACATTTGAGAAAGCTTGCCCTC
pCMV-Flag-PA-K262R	Mutation	F:TTGAACCTTTTTTGAGATCAACACCACGACC R:CTCAAAAAAGGTTCAATTCTAGCATT
pCMV-Flag-PA-K281R	Mutation	F:GTTCTCAGCGGTCCAGATTCCTGCTGATGGA R:CTGGACCGCTGAGAACAGGGAGGCCC
pCMV-Flag-PA-K309R	Mutation	F:TATATGATGCAATCAGATGCATGAGAACATT R:CTGATTGCATCATATAGCGGTATCCC
pCMV-Flag-PA-K339R	Mutation	F:ATCTTCTGTCATGGAGGCAAGTACTGGCAGA R:CTCCATGACAGAAGATAATTTGGATT
pCMV-Flag-PA-K353R	Mutation	F:TTGAGAATGAGGAGAGAATTCCAAGGACTAA R:CTCTCCTCATTCTCAATGTCCTGCAG
pCMV-Flag-PA-K391R	Mutation	F:ATGTAGGCGATTTGAGGCAATATGATAGTGA R:CTCAAATCGCCTACATCTTTACAGTC
pCMV-Flag-PA-K536R	Mutation	F:GACTTGAACCACACAGATGGGAGAAGTACTG R:CTGTGTGGTTCAAGTCTTGGGTCAGT
pCMV-Flag-PA-K605R	Mutation	F:CCTCTGTCAAGGAGAGAGACATGACCAAAGA R:CTCTCCTTGACAGAGGACTCAGCTTC
pCMV-Flag-PA-K609R	Mutation	F:AGAAAGACATGACCAGAGAGTTCTTTGAGAA R:CTGGTCATGTCTTTCTCCTTGACAGA
pCMV-Flag-PA-K615R	Mutation	F:AGTTCTTTGAGAACAGATCAGAAACATGGCC R:CTGTTCTCAAAGAACTCTTTGGTCAT
pCMV-Flag-PA-K626R	Mutation	F:TTGGAGAGTCCCCCAGAGGAGTGGAGGAAGG R:CTGGGGGACTCTCCAACGGGCCATGT
pCMV-Flag-PA-K635R	Mutation	F:AAGGTTCCATTGGGAGGGTCTGCAGAACTTT R:CTCCCAATGGAACCTTCCTCCACTCC
pCMV-Flag-PA-K643R	Mutation	F:GAACTTTATTGGCAAGGTCGGTATTCAACAG R:CTTGCCAATAAAGTTCTGCAGACCTT
pCMV-Flag-PA-K664R	Mutation	F:CAGCTGAATCAAGAAGACTGCTTCTTATCGT R:CTTCTTGATTCAGCTGAAAATCCTTC
siNC	Knock down	UUCUCCGAACGUGUCACGUTT
siDCAF7#1	Knock down	GCGUCUAUCCAGACCUACUTT
siDCAF7#2	Knock down	GGUGGAUCCUUAUCUUUUATT
sgRNA#1	Knock out	GCGGTGACTATCTCCGTGTG
sgRNA#2	Knock out	AGCCTGGTGGTCATCCGCTA
sgRNA#3	Knock out	AGTCACCGCTTGTTGCCAGT
PR8 NP-vRNA	RT	GGCCGTCATGGTGGCGAATGAATGGACGGAGAACAAGGATTGC
PR8 NP-mRNA	RT	CCAGATCGTTCGAGTCGTTTTTTTTTTTTTTTTTCTTTAATTGTC
PR8 NP-cRNA	RT	GCTAGCTTCAGCTAGGCATCAGTAGAAACAAGGGTATTTTTCTTT
H3N2 NP-vRNA	RT	CTCACCGAGTGACATCAACATCATG
PR8 NP-vRNA	qPCR	F:GGCCGTCATGGTGGCGAAT R:CTCAATATGAGTGCAGACCGTGCT
PR8 NP-mRNA	qPCR	F:CTCAATATGAGTGCAGACCGTGCT R:CGATCGTGCCCTCCTTTG
PR8 NP-cRNA	qPCR	F:GCTAGCTTCAGCTAGGCATC R:CGATCGTGCCCTCCTTTG
H3N2 NP-vRNA	qPCR	F:ATCAGACCGAACGAGAATCCAGC R:GGAGGCCCTCTGTTGATTAGTGT
Human GAPDH	qPCR	F:AGGTCGGAGTCAACGGATTT R:TGACGGTGCCATGGAATTTG
Human DCAF7	qPCR	F:CGCCATCTAGAACACAGCAC R:GCCATTGACACATGCTCGAT
Mouse Actin	qPCR	F:CTAAGGCCAACCGTGAAAAG R:ACCAGAGGCATACAGGGACA
Mouse DCAF7	qPCR	F:GGACTGTGTACGCCATGAAC R:CTTTGTGGTAGGGTACGGGT
Human DCAF2	qPCR	F:CCAGTATCTCAGAGCCTCCG R:TGGATTCTCAGCCTTCCGTT
Human DCAF3	qPCR	F:AATTTACCCAGACCCAGCGA R:GACCCTGGTTGTCTGAGGAA
Human DCAF5	qPCR	F:ACAAGCAGCCAGGATGTACT R:CAAAGAAGGCCATCATCCGG
Human DCAF6	qPCR	F:CATACCCAGCAACAGCCTTC R:GTTTGGGGCTCAGCTTTTCA
Human DCAF9	qPCR	F:ACCTATGTGACCTTCAGCCC R:GACATGTCCCCTACTCTCCG
Human DCAF11	qPCR	F:TGGCCAGCAGTTCATCTACA R:TGGCATGTCATCCTGGAAGT
Human DCAF13	qPCR	F:GAAGATGCTGAGCCGGAATC R:GGACAGTAGCCAGCTTCTCT
Human DCAF14	qPCR	F:ACTCAGCAAGCACCTCATCT R:CCTGGTTTGCTTGCTGACTT
